# Clustering effects on the reactivity of alkoxy radicals: rate coefficients of ^3^(RO⋯OR) complexes accounting for multiple conformers

**DOI:** 10.1039/d5cp04635a

**Published:** 2026-04-17

**Authors:** Hongye Fraise Zhao, Lauri Franzon, Severi Juttula, Robert Skog, Nanna Myllys, Theo Kurtén

**Affiliations:** a Department of Chemistry, University of Helsinki, A.I. Virtasen aukio 1 (Chemicum) 00560 Helsinki Finland theo.kurten@helsinki.fi +358 (0)294150289

## Abstract

Atmospheric peroxy (RO_2_˙) and alkoxy (RO˙) radical species are crucial intermediates in the formation of secondary organic aerosol (SOA). Recent computational work has demonstrated that recombination reactions of peroxy radicals (RO_2_˙ + RO_2_˙) proceed through triplet complexes consisting of two alkoxy radicals (^3^(RO⋯OR)). To understand how peroxy recombination reactions branch into different product channels, it is thus necessary to thoroughly investigate the reactions of these triplet alkoxy complexes. Although the reactions of free alkoxy radicals have been extensively studied, the reactivity of triplet alkoxy complexes remains relatively less explored. In this study, we have therefore developed a systematic conformer sampling workflow for ^3^(RO⋯OR) and applied it to four typical alkoxy systems (AceO, β-ISOPO, PhCH_2_O, and PhC(O)O). Rate coefficients (*k*) of key reactions have been calculated using multi-conformer transition state theory (MC-TST) and lowest-conformer transition state theory (LC-TST), allowing the quantitative evaluation of conformer effects. Our results demonstrate that the presence of the other RO˙ in the complex has a noticeable effect on *k* values. For the β-ISOPO system in particular, the predicted β-scission *k* values in the ^3^(RO⋯OR) complex are over 100 times higher than those of the free radicals.

## Abstract 2

A systematic conformer sampling workflow reveals novel pathways and enhanced β-scission in alkoxy complexes (^3^(RO⋯OR)) with atmospheric significance.

## Graphical abstract

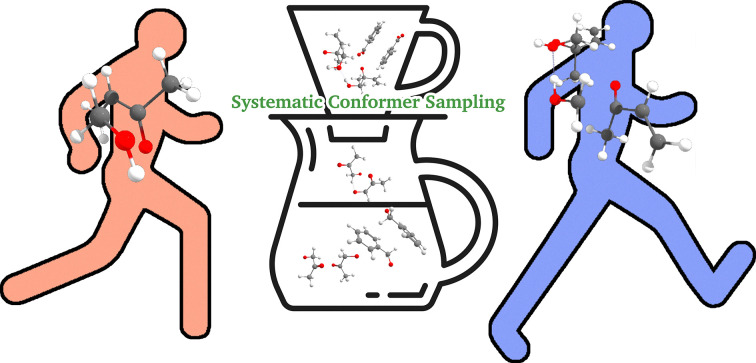

## 1 Introduction

Climate change and air pollution have become major challenges in current times. As a pollutant, small atmospheric particles account for around 10 million global deaths and 10^8^ disability-adjusted life years annually.^1,2^ On the other hand, atmospheric aerosols can cool the climate through cloud formation and scattering.^3^ Sustainable development actions have significantly reduced the emission of inorganic species (*e.g.* NO_*x*_, SO_2_, *etc.*), initiating a shift in aerosol compositions: before those actions, atmospheric aerosols were dominated by primary inorganic aerosols, while after those actions, secondary organic aerosol (SOA) particles have become more prominent.^4^

**Fig. 1:**
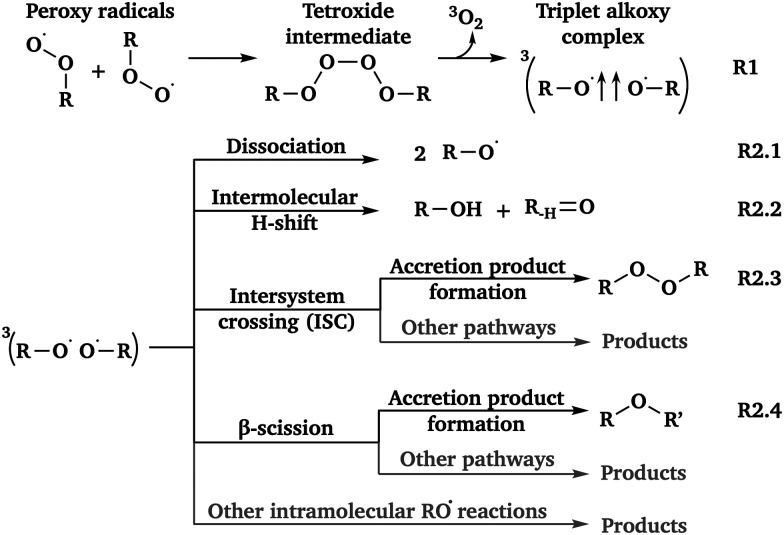
The known product channels of peroxy recombination reactions.

Volatile organic compounds (VOCs) are a major source of SOA, and come from various biogenic (*e.g.*, isoprene molecules released by vegetation)^5^ and anthropogenic (*e.g.*, fuel combustion, usage of organic solvents)^6^ emissions. After emission, VOCs are oxidized and converted into peroxy radicals (RO_2_˙). Depending on its molecular structure, RO_2_˙ can undergo both unimolecular reactions, such as H-shifts and ring closures, and bimolecular reactions with species, including NO_*x*_, HO_*x*_ and RO_2_˙.^7^ Among these channels, we are especially interested in the peroxy recombination reaction ((R1) in Fig. 1), as some subsequent channels can generate accretion products^8^ (*e.g.* (R2.3) and (R2.4) in Fig. 1), which are critical for SOA formation. Compared to the initial VOCs, accretion products have a higher number of carbon atoms and lower volatility,^9^ allowing them to cluster very efficiently.

As shown in Fig. 1, the peroxy recombination reaction proceeds *via* a tetroxide intermediate, and generally††According to the experimental results of Murphy *et al.*,^10^ for certain peroxy radicals such as HOCH_2_CH_2_OO˙, the recombination reaction might also proceed through a ^1^(RO⋯O_2_⋯OR) complex and produce HO_2_˙. The reaction can be summarized as follows: RO_2_˙ + RO_2_˙ → ^1^(RO⋯O_2_⋯OR) → R_–H_=O + HO_2_˙ + RO˙. leads to a triplet alkoxy complex (^3^(RO⋯OR)) after releasing one ^3^O_2_ molecule.^11,12^ The ^3^(RO⋯OR) complex consists of two alkoxy radicals (RO˙) and can undergo various reactions. Therefore, the branching of peroxy recombination channels reflects different fates of ^3^(RO⋯OR).

So far, four reaction channels of ^3^(RO⋯OR) have been discovered: dissociation^13^ ((R2.1) in Fig. 1), intermolecular H-shift^14,15^ ((R2.2) in Fig. 1), intersystem crossing (ISC)^16^ to the singlet surface ((R2.3) in Fig. 1), and β-scission of one of the alkoxy radicals^17,18^ ((R2.4) in Fig. 1). Rate coefficients (*k*) reported for these channels vary widely with both the structure of ^3^(RO⋯OR) and the calculation method. Nevertheless, all four channels share a common feature: they can proceed very rapidly, with estimated *k*(298 K) values often exceeding 10^9^ s^−1^.^14,15,17^

In addition to β-scissions, other intramolecular reactions may also take place depending on the structure of the reactive RO˙. These intramolecular reactions include CO_2_ elimination in acyloxy radicals,^19^ intramolecular H-shifts^20,21^ and epoxide formation.^22,23^ We expect that any sufficiently fast intramolecular RO˙ reaction^24^ can also occur in ^3^(RO⋯OR)—but the in-complex rate is likely to differ from that of the free-radical reaction.

Among the reaction channels of ^3^(RO⋯OR), ISC and β-scission are of special importance, as they may lead to recombination processes that form accretion products. In general, the ester or ether products from β-scission (ROR′) are expected to be more stable and, therefore, longer-lived than the peroxide products from ISC (ROOR).^17^ However, note that neither ISC nor β-scission reactions guarantee the accretion product formation, as other competing processes may intervene before recombination. For example, H-shift and dissociation reactions can take place in ^1^(RO⋯OR) (from ISC)^25^ or ^3^(R′⋯OR⋯CH_2_O) (from β-scission) complexes. Additionally, the ROR′ formation after β-scission probably requires a subsequent ISC.^17^

Given the importance of atmospheric ^3^(RO⋯OR) complexes, a natural progression is to investigate the rate coefficients of their different reactions. Numerous experimental studies have probed the final product distribution of peroxy radical recombination. For example, Frandsen *et al.*^18^ recently studied the ozonolysis of tetramethyletylene (TME), where the dominant first-generation RO_2_˙ is the acetonyl peroxy radical, CH_3_C(O)CH_2_OO˙ (AceOO˙). Products with the elemental composition of C_6_H_10_O_4_ were abundantly observed, suggesting ISC as the major channel. On the other hand, Peräkylä *et al.*^17^ detected C_19_ esters as the primary accretion product of α-pinene (C_10_) ozonolysis, implying that β-scission dominates the accretion product formation.

Although experiments can probe the final product distribution of the RO_2_ + RO_2_ reactions and even have recently captured the tetroxide intermediate in the gas phase (observed as R_2_O_4_(H_2_O)H^+^),^26^ current techniques are still unable to detect ^3^(RO⋯OR) directly, as this complex is too short-lived for spectroscopic detection or isolation. Alternatively, theoretical methods can reveal the structures of key intermediates and estimate the stepwise reaction rates.

Previous theoretical studies^14,15^ have shown the difficulties of studying the in-complex reactions: (1) the ^3^(RO⋯OR) complex, an open-shell species with two unpaired electrons, is different from the benchmarking species used by major computational chemistry methods, up to and including state-of-the-art methods such as coupled cluster. It is noteworthy that the training sets of the xTB methods, which are commonly used for semi-empirical conformer sampling, are primarily closed-shell species.^27–29^ (2) The structural variety of ^3^(RO⋯OR) conformers affects the accuracy of the predicted thermodynamic and kinetic parameters. By definition, each conformer corresponds to a distinct potential energy minimum.^30^ Energetically low-lying conformers influence the rate coefficient as their Boltzmann population is non-zero.^31,32^ Previous computational studies have suggested that ignoring conformers other than the global minimum may lower the accuracy of the rate coefficient prediction, especially at higher temperatures relevant to combustion.^31,33^ Furthermore, compared to RO˙, a larger number of conformers can be expected for ^3^(RO⋯OR) due to intermolecular interactions.

Based on the discussions above, to compute the reaction rate coefficients of ^3^(RO⋯OR) more accurately, a systematic conformer search workflow is required. In this study, we improve the conformer sampling workflow reported by Hasan *et al.*^14,15^ and Møller *et al.*,^31^ with a special focus on intermolecular H-shift reactions. Since these reactions involve both RO˙ in the ^3^(RO⋯OR) complex and certain computational tools (*e.g.* GOAT) had not been developed at that time, previous studies^14,15^ encountered challenges to sample the transition state (TS) conformers of intermolecular H-shift reactions comprehensively. Then, we apply our updated workflow to four ^3^(RO⋯OR) systems corresponding to representative RO_2_˙ + RO_2_˙ pairs in the atmosphere. We determine the *k* values of β-scission, intermolecular H-shift and other selected reactions, such as RO˙ addition to a double bond (R3.2.2(a) and (b) in Fig. 3) and epoxy formation–decomposition (R3.3.2(1) and (2) in Fig. 4). In addition to investigating the relevance of these novel pathways, our main purpose is to assess how clustering with another RO˙ affects the reactivity of alkoxy radicals, which is achieved by comparing the free-radical and in-complex *k* values.

This study does not include the rate coefficients of dissociation and ISC channels. For the dissociation channel, *k* strongly depends on the binding energy (*D*) of ^3^(RO⋯OR),^13^ and the *D* values are reported in Table S2 of the supplementary information (SI) (relevant notations are defined in Table S1). For the ISC channel, determining both the ISC rate and the post-ISC product distribution requires additional calculations on the highly multireference open-shell singlet surface, which is beyond the scope of this work.

The names and RO˙ molecular formulas of the four systems are listed in Table 1. Each system includes both free radicals and triplet complexes. The simplified notations for ^3^(RO⋯OR) are similar to those of their corresponding RO˙.

**Table 1:** Systems involved in this study and the names used to refer to them throughout this paper

Simplified notation in this paper	Full name	Molecular formula of RO˙	Structure of RO˙
AceO radical or AceO˙	Acetonyl alkoxy radical	C_3_H_5_O_2_	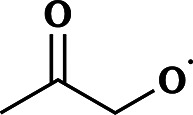
β-ISOPO radical or β-ISOPO˙	(*S*)-1-Methyl-1-vinyl-2-hydroxyethyl alkoxy radical	C_5_H_9_O_2_	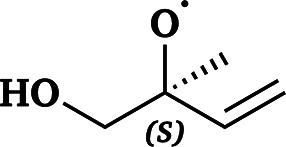
PhCH_2_O radical or PhCH_2_O˙	Benzyl alkoxy radical	C_7_H_7_O	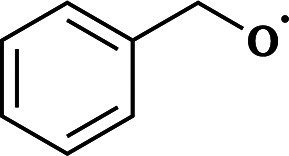
PhC(O)O radical or PhC(O)O˙	Benzyl acyloxy radical	C_7_H_5_O_2_	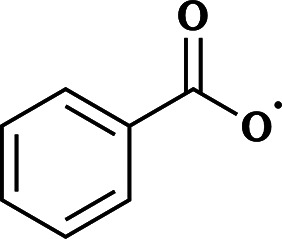

These four systems were selected based on experimental evidence and atmospheric importance: AceO˙ can be formed in tetramethylethylene (TME) ozonolysis, and several experimental studies^18,34,35^ have investigated its product distribution. β-ISOPO˙, as the name implies, can be generated in the ˙OH-oxidation processes of isoprene.^36^ Since stereochemistry is not the focus of this study, we selected only (*S*)-enantiomers of β-ISOPO˙ for simplicity. PhC(O)O˙ and PhCH_2_O˙ were selected as representative aromatic alkoxy radicals as they are formed during the oxidation of toluene. In brief, Cl˙, ˙OH or NO_3_˙ initiate a methyl H-abstraction reaction from toluene,^37^ followed by O_2_ addition to form PhCH_2_OO˙. Recombination reactions of PhCH_2_OO˙ then generate the corresponding alkoxy radical, PhCH_2_O˙, as well as benzaldehyde (PhCHO).^38^ Subsequently, PhCHO can be oxidized into PhC(O)O˙.^39^

## 2 Computational details

### 2.1 Notations and computational chemistry programs applied in this study

For clarity and succinctness, Table 2 summarizes the simplified notations of the types of reactants and transition states, computational methods and energy terms used in this study. Detailed discussion of computational methods is provided in Sections 2.2–2.4. A schematic representation of the conformer sampling workflow is reported in Fig. S1.

**Table 2:** Simplified notations of conformers, computational methods and energy terms used in this study

Simplified notation in this paper	Detailed descriptions
Reactant monomer	An isolated RO˙ radical.
TS monomer	A transition state conformer of intramolecular RO˙ reactions.
Reactant dimer	A triplet alkoxy complex, ^3^(RO⋯OR).
TS H-shift conformer	A transition state conformer of the intermolecular H-shift reaction of ^3^(RO⋯OR), which involves both RO˙ in the complex.
TS dimer	A transition state conformer of other intramolecular reaction channels of ^3^(RO⋯OR), which takes place within one RO˙ of the complex.
B3LYP	B3LYP/ma-def2-SVP.
ωB97X-D3 (Grid2)	ωB97X-D3/ma-def2-TZVP, with the default grid size of ORCA 6.0.1.
ωB97X-D3 (Grid3)	ωB97X-D3/ma-def2-TZVP, with the largest predefined grid size of ORCA 6.0.1.
DLPNO-CCSD(T)-F12	UHF-DLPNO-CCSD(T)-F12/cc-pVDZ-F12, with the keyword “tightPNO” and “tightSCF”, cc-pVDZ-F12-CABS as the auxiliary basis set, and aug-cc-pVDZ/C as the RI approximation basis set.
*f*(DFT)	Values calculated at the ωB97X-D3/ma-def2-TZVP (DefGrid3) level of theory, where *f* can be an energy term (*E*) or rate coefficient (*k*).
*f*(CC)	Values calculated at the UHF-DLPNO-CCSD(T)-F12/cc-pVDZ-F12 level of theory, where *f* can be *E* or *k*.
*E*_el_	Electronic energy.
*E*_elzc_	Zero-point corrected electronic energy.
*G*	Gibbs free energy.
*D*	Dissociation energy of a complex.
*E*_sp_	Single-point energy.
Δ_b_*E*	The energy barrier of a reaction (Δ_b_*E* = *E*_TS_ − *E*_reactant_), which reflects the kinetic feasibility of the reaction.
Δ_r_*E*	The reaction energy (Δ_r_*E* = *E*_Product_ − *E*_Reactant_), which reflects the thermodynamic feasibility of the reaction.

The initial structures of isolated RO˙ were built using SPARTAN′24.^40^ Reactant and transition state (TS) dimers were generated using the artificial bee colony algorithm for cluster global optimization (ABCluster) 3.3 program.^41,42^ Transition state structures of intermolecular H-shift reactions (TS H-shift) were generated using the global optimization algorithm (GOAT)^43^ implemented in ORCA 6.0.1.^44^ Semi-empirical calculations were performed using XTB 6.7.1.^45,46^ Calculations at higher level of theories, including density functional theory (DFT) and coupled cluster methods, were performed using ORCA 6.0.1.^44^ Filtering and analysis of the results were done using the Jammy Key for Configurational Sampling (JKCS) 2.1 program.^47–49^

### 2.2 Method for rate coefficient calculation and relevant energy barriers

Earlier studies reported that β-scission and intermolecular H-shift reactions of ^3^(RO⋯OR) proceed through a single transition state,^14,15^ and can thus be treated as elementary reactions.^50^ In addition, reactions taking place within a ^3^(RO⋯OR) complex do not involve collisions or exchanges with other molecular entities, and can therefore be treated as unimolecular elementary reactions. These reactions are further categorized as follows: if the reaction involves only one RO˙ in the ^3^(RO⋯OR) complex, it is a unimolecular intramolecular reaction. In contrast, if the reaction involves both RO˙ in the complex, it is a unimolecular intermolecular reaction. The first category includes β-scission, epoxy formation and decomposition, while the second category includes intermolecular H-shift and RO˙ addition to the double bond.

Therefore, reaction rate coefficients in this study are calculated using the elementary transition state theory (TST).^51,52^ Although absolute *k* values predicted by elementary TST are often inaccurate, the relative rates are more reliable (*e.g.*, the 
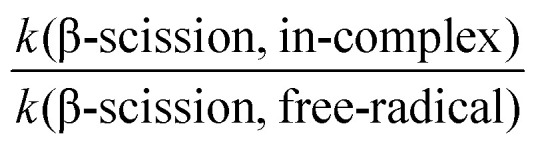
 factor, which is further discussed in Section 3.5). In addition, according to Hasan *et al.*,^14^ variational effects are unlikely to be a major source of error even for rapid reactions in alkoxy complexes. The differences between their H-shift rate coefficients computed using elementary and variational TST are generally within a factor of two,^14^ suggesting that elementary TST is capable of describing such systems, and consequently, sufficient for this study.

In practice, elementary TST is applied in two forms when calculating *k*:^31^ multi-conformer TST (MC-TST),^31,53^ which accounts for contributions from multiple conformers (within an energy cutoff) in eqn (1); or lowest-conformer TST (LC-TST),^31^ which includes only the conformer with the lowest (free) energy value, and thus with the highest Boltzmann probability, in eqn (2).1
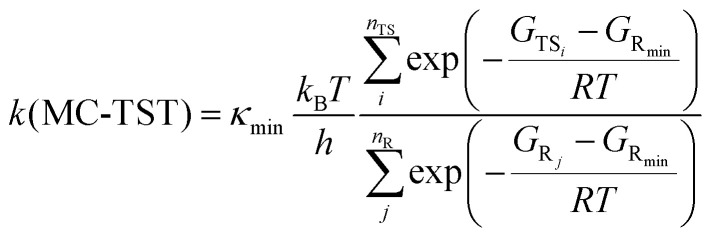
2
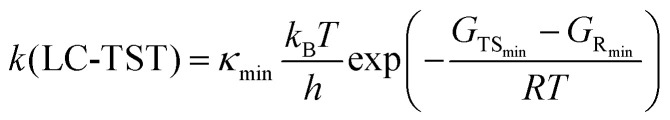


In eqn (1) and (2), *κ*_min_ is the tunneling coefficient, *T* is the temperature, *k*_B_ is the Boltzmann constant, *h* is the Planck constant, *R* is the gas constant, *G*_TS_ is the Gibbs free energy value of the transition state and *G*_R_ is the Gibbs free energy value of the reactant. The subscripts indicate the following information: “min” is for the lowest-energy conformer, “*i*” is for any TS conformer, and “*j*” is for any reactant conformer.

As Møller *et al.*^31^ reported, one *κ* value is often sufficient to represent the tunneling effect for all conformers of the same reaction. This coefficient corresponds to the tunneling coefficient of the lowest-energy TS conformer,^31^ denoted *κ*_min_. In practice, the *κ*_min_ value was calculated using the Eckart tunneling method,^54^ which requires the forward and backward barrier heights and imaginary frequency (*ω*_i_) of the TS on the potential energy surface (PES). The PES was obtained using the intrinsic reaction coordinate (IRC)^55^ approach in ORCA 6.0.1. The IRC endpoints were then optimized, and their zero-point corrected electronic energy (*E*_elzc_) values were used to compute the barrier heights for the *κ*_min_ calculation.^31,32^

### 2.3 Conformer sampling of free-radical reactions

For each reaction channel, a single RO˙ structure (reactant monomer) from SPARTAN was optimized at two density functional theory (DFT) levels in ORCA: first at the B3LYP^56,57^/ma-def2-SVP^58,59^ level and then at the ωB97X-D3^60,61^/ma-def2-TZVP^58,59^ level. The choice of B3LYP and ωB97X-D3 functionals was made based on previous studies on ^3^(RO⋯OR) complexes,^14,15,17,21^ as well as benchmarking work on general chemical databases^62^ and atmospheric molecular clusters.^63^ Tests of different basis sets on ^3^(AceO⋯OAce)^64^ and small gas-phase clusters^65^ indicate that ma-def2-SVP and ma-def2-TZVP basis sets are sufficiently accurate for the systems studied in this paper with good computational efficiency.

After optimization at the B3LYP level, the reactant monomer was optimized at the ωB97X-D3 level in two subsequent steps: first with the default grid size (no additional keywords required, or specified by the keyword “DefGrid2”)^44,66^ and then with the largest predefined grid size in ORCA 6.0.1 (invoked by the keyword “DefGrid3”).^66,67^ These two steps at ωB97X-D3 level will be referred to as “ωB97X-D3 (Grid2)” and “ωB97X-D3 (Grid3)” throughout this paper. Grid3 was applied to eliminate the imaginary frequencies caused by numerical noise.^68^ If there were still small imaginary frequencies after optimizations with Grid3, approaches such as subtle displacement along the vector or applying tighter convergence criteria would be applied.

The TS monomer was determined by relaxed surface scans at the B3LYP level of theory. The TS structure from scans were then optimized at the B3LYP, ωB97X-D3 (Grid2) and ωB97X-D3 (Grid3) levels, similar to its corresponding reactant monomer.

Based on the optimized reactant and TS monomer structures, conformer searches were performed using GOAT with the GFN2-xTB^28,45^ method in ORCA, with an energy cutoff of 15 kcal mol^−1^.‡‡The conformer search will stop going uphill if *E*(new conformer) − *E*(initial conformer) > 15 + 3*N*_atom_ kcal mol^−1^, where *N*_atom_ is the number of atoms in the conformer. This is invoked by the keyword “MaxEn 15.0”.^43^ Conformers generated by GOAT were then optimized at the B3LYP level, followed by filtering based on their uniqueness and relative energy values. Detailed selection criteria are:

• Any two selected conformers must differ by more than 0.01 Å in gyration radius (Δ*R*_g_ > 0.01), 0.001 Hartree in electronic energy (Δ*E*_el_ > 0.001 Hartree), and 0.1 Debye in dipole moment (Δ*p* > 0.1 Debye).^14,49^ This criterion, which is also the default setting in JKCS 2.1, will hereafter be referred to as the “uniqueness filter” or “filtered by uniqueness”.

• The Gibbs free energy value of every selected conformer must not be more than 5 kcal mol^−1^ (ref. 14 and 31) above the global minimum (*G* − *G*_min_ < 5 kcal mol^−1^). This criterion will be referred to as “*G*_cutoff_ = 5 kcal mol^−1^” hereafter. Other energy-based filters will also be written in the form of “*E*_energy type, cutoff_ = *x* kcal mol^−1^”.

B3LYP-optimized and filtered conformers were further optimized at the ωB97X-D3 (Grid2) level, and the optimized structures were filtered by uniqueness. Following this, the conformers were optimized at the ωB97X-D3 (Grid3) level, and then filtered by uniqueness and *G*_cutoff_ = 2 kcal mol^−1^.

Conformers satisfying all the criteria discussed above were forwarded to the next step. To improve the accuracy of relative energy predictions, single-point energy calculations on top of the ωB97X-D3 (Grid3) optimized structures were performed using the following coupled cluster method: UHF^69^-DLPNO-CCSD(T)^70^-F12^71^/cc-pVDZ-F12,^72^ with the keywords “tightPNO”^73^ and “tightSCF”, cc-pVDZ-F12-CABS^72^ as the auxiliary basis set, and aug^74^-cc-pVDZ^75^/C as the RI approximation basis set. This method was suggested by our benchmarking results and previous studies on atmospheric molecular clusters.^63,76^ Coupled cluster calculations were only carried out on lowest-energy conformers (*G*_min_), as suggested by previous studies.^14,15,31,32^

Following the DLPNO-CCSD(T)-F12 calculations, energy corrections were computed as *E*_sp_(CCSD(T)) − *E*_el_(DFT) for the lowest-energy reactant and TS monomers, respectively, and applied to other conformers of the same type. The coupled cluster-corrected *G*_R_ and *G*_TS_ values were applied to calculate the *k*(CC) of free-radical reactions.

Finally, *k*(DFT) and *k*(CC) were computed using both MC-TST (eqn (1)) and LC-TST (eqn (2)) methods to evaluate the effects of coupled-cluster corrections and contributions from multiple conformers. Consequently, four *k* values were reported for each reaction channel.

### 2.4 Conformer sampling of in-complex reactions

#### 2.4.1 Conformer sampling of reactant dimers

Based on the reactant monomers from Section 2.3, reactant dimers were generated using ABCluster 3.3, executed through JKCS2.1. Monomers were treated rigidly (*i.e.*, with fixed internal coordinates)^42^ and placed against each other with a CHARMM36^77,78^-based force field. ABCluster requires three types of input data:^41,42^ monomer coordinates (in the .xyz format), atom-wise partial charges and force field parameters (*ε* and *σ*). Partial charges were calculated using the natural bond orbital (NBO) 7.0.10^79,80^ program interfaced to ORCA 6.0.1. The *ε* and *σ* values were generated using the “topgen” module of ABCluster and then examined and adjusted by the authors when necessary. With the input data above, ABCluster was invoked by the following command: JKCS2_explore -pop 1280 -gen 320 -lm 100 -exploded -repeat 10. This command would guarantee that the initial number of dimers generated for each monomer combination was 1000,§§For example, if there are two conformers of AceO˙, the number of possible ^3^(AceO⋯OAce) combinations will be three, and the ABCluster step will generate 3000 initial structures of ^3^(AceO⋯OAce). as previous studies suggested.^15,81^

Reactant dimers were collected and optimized using the GFN1-xTB^27,45^ method with necessary constraints. For atmospheric molecular clusters, the GFN1-xTB method was reported to have better agreement with the DFT energy values than GFN2-xTB.^81,82^ In addition, GFN1-xTB showed less problems than GFN2-xTB during the geometry optimization of alkoxy dimers. GFN1-optimized structures were then filtered by uniqueness and *E*_el, cutoff_ = 15 kcal mol^−1^.^15^

The filtered conformers were then optimized at the B3LYP level in ORCA. Based on our benchmarking results, applying the *E*_el_ cutoff of 5 kcal mol^−1^ was equivalent to applying the *G* cutoff of 5 kcal mol^−1^. Thus, frequency calculations were omitted at this step to reduce computational cost. After B3LYP optimization, reactant dimers were filtered by uniqueness and *E*_el, cutoff_ = 5 kcal mol^−1^.^15,31^

Subsequently, reactant dimers were optimized at the ωB97X-D3 level in ORCA. Geometry optimizations and frequency calculations were first performed with Grid2 and filtered by uniqueness. Similar calculations were then performed with Grid3, with special attention to convergence and frequencies, and the final structures were filtered by uniqueness.

The reactant dimer with the lowest *G* value at the ωB97X-D3 (Grid3) level was selected for the coupled cluster calculation. Its *E*_sp_ was calculated using the DLPNO-CCSD(T)-F12 method, and the energy correction (see Section 2.3) was applied to other reactant dimers. The coupled cluster-corrected *G* values of the reactant dimers were substituted into the *G*_R_ terms of eqn (1) and (2).

#### 2.4.2 Conformer sampling of TS H-shift conformers

As discussed in Section 2.4.1, ABCluster 3.3 requires “isolated” monomers as input to generate the corresponding dimers. However, the TS H-shift conformer resembles more a connected moiety (two RO˙ connected through a C–H⋯O structure) rather than two separate monomers. Thus, ABCluster is not suitable for building initial TS H-shift structures. Alternatively, generation and semi-empirical optimization of TS H-shift conformers were performed using GOAT, with the GFN1-xTB method and an energy cutoff of 15 kcal mol^−1^.

TS H-shift conformers from GOAT were then optimized using the B3LYP method. A constrained optimization without frequency calculation was first performed. The constrained optimization step fixes the distance between the two atoms strongly involved in the TS, while relaxing and optimizing the rest of the structure.^83^ For the alkoxy systems studied, this step can reduce the computational cost and allow the following TS optimization to converge more easily. Each imaginary frequency was checked carefully to ensure it corresponded to the expected vibrational mode. Conformers that converged with correct vibrational modes at the B3LYP level were filtered by uniqueness and *G*_cutoff_ = 5 kcal mol^−1^.

Following that, TS H-shift conformers were optimized at the ωB97X-D3 level in three steps: constrained optimization, TS optimization with Grid2, and TS optimization with Grid3. Frequencies were carefully examined, and de-noise approaches described in Section 2.3 were applied if necessary. A uniqueness filter was applied both before and after the ωB97X-D3 (Grid3) optimization.

Finally, a single point calculation of the TS H-shift conformer at the DLPNO-CCSD(T)-F12 level was performed on top of the ωB97X-D3 (Grid3) optimized structure with the lowest *G* value. Energy corrections were calculated as discussed in Section 2.3. The coupled cluster-corrected *G* values of TS H-shift conformers were then substituted into the *G*_TS_ terms of eqn (1) and (2). Together with the *G*_R_ terms from Section 2.4.1, these values were applied to calculate the *k*(CC) of the intermolecular H-shift reaction. In addition, the *k*(DFT) values were also calculated as described in Section 2.3.

#### 2.4.3 Conformer sampling of TS dimers

For other intramolecular reaction channels of ^3^(RO⋯OR), their TS conformers behave more like a complex consisting of two separate RO˙ moieties, similar to the reactant dimers described in Section 2.4.1. Therefore, ABCluster can be used to generate these TS dimers with a suitable input (one reactant monomer and one TS monomer). Semi-empirical optimizations were performed using the GFN1-xTB method. After applying the uniqueness and *E*_el, cutoff_ = 15 kcal mol^−1^ filters, TS dimers were optimized at the B3LYP level in two steps: constrained and TS optimization. Geometry convergence and frequencies were carefully examined, followed by the uniqueness and *G*_cutoff_ = 5 kcal mol^−1^ filters.

After B3LYP optimizations and filtering, the TS dimers were optimized at the ωB97X-D3 level in three steps, as described in Section 2.4.2. A uniqueness filter was applied both before and after the ωB97X-D3 (Grid3) optimization. Frequencies were examined as mentioned in Section 2.4.2, and when required, de-noise approaches (see Section 2.3) were applied. Thereafter, the *E*_sp_ values were calculated at the DLPNO-CCSD(T)-F12 level, and energy corrections were applied as mentioned in Section 2.3. The coupled cluster-corrected *G* values of TS dimers were substituted into the *G*_TS_ terms of eqn (1) and (2). Together with the *G*_R_ terms from Section 2.4.1, these values were employed to compute the *k*(CC) of other intramolecular ^3^(RO⋯OR) reactions. Additionally, the *k*(DFT) values were also calculated as described in Section 2.3.

## 3 Results and discussion

This section first presents the number of conformers for each alkoxy system after sampling (Table 3) and the calculated *k* values using different methods (Table 4). Further discussion on each system is provided in the following subsections. Multireference and spin contamination diagnostics, conformer-wise relative energy values (including conformers from sampling and IRC endpoints) and imaginary frequencies that correspond to vibrational modes of the transition state are reported in Tables S3–S10.

**Table 3:** Final conformers for *k* calculation after ωB97X-D3 (Grid3) optimization and filtering

System	Conformer type	Number
AceO	Reactant monomer	2
	TS monomer (β-scission)	2
	Reactant dimer	7
	TS dimer (β-scission)	3
	TS H-shift conformer	11

β-ISOPO	Reactant monomer	8
	TS monomer (β-scission, hydroxymethyl side)	6
	Reactant dimer	25
	TS dimer (β-scission, hydroxymethyl side)	26

PhCH_2_O	Reactant monomer	1
	TS monomer (epoxy formation)	1
	Epoxy radical	1
	TS monomer (epoxy decomposition)	1
	Reactant dimer	3
	TS dimer (epoxy formation)	7
	Epoxy–PhCH_2_O complex	12
	TS dimer (epoxy decomposition)^a^	12
	TS H-shift conformer	3

PhC(O)O	Reactant monomer	1
	TS monomer (β-scission)	1
	Reactant dimer^a^	9
	TS dimer (β-scission)^a^	7

a The total number of conformers was counted after excluding the high-energy conformers which had a small imaginary frequency that could not be eliminated (see Sections 3.3 and 3.4 for detailed discussion).

**Table 4:** Rate coefficients of four alkoxy systems at 298.15 K. The *k* values were calculated at the ωB97X-D3 (Grid3) (labeled “DFT”) and DLPNO-CCSD(T)-F12 (labeled “CC”) levels using LC-TST (labeled “LC”) and MC-TST (labeled “MC”) methods, and are reported in s^−1^

System	Reaction type	*κ*_min_ (DFT)	*κ*_min_ (CC)	*k*_LC_ (DFT)	*k*_LC_ (CC)	*k*_MC_ (DFT)	k_MC_ (CC)
AceO	β-Scission, free-radical	1.0	1.0	2.33 × 10^8^	3.57 × 10^9^	2.33 × 10^8^	3.56 × 10^9^
	β-Scission, in-complex	1.0	1.0	1.65 × 10^9^	1.54 × 10^10^	6.40 × 10^8^	5.99 × 10^9^
	Intermolecular H-shift	1.7	1.7	8.91 × 10^6^	6.22 × 10^5^	1.29 × 10^7^	8.98 × 10^5^

β-ISOPO^a^	β-Scission, free-radical	1.2	1.2	2.91 × 10^9^	7.44 × 10^8^	3.70 × 10^9^	9.47 × 10^8^
	β-Scission, in-complex	1.1	1.1	7.49 × 10^11^	6.46 × 10^11^	3.73 × 10^11^	3.22 × 10^11^

PhCH_2_O	Epoxy formation, free-radical^b^	1.3	1.3	1.19 × 10^3^	1.59 × 10^4^	1.19 × 10^3^	1.59 × 10^4^
	Epoxy formation, in-complex	1.2	0.9	2.93 × 10^3^	2.33 × 10^4^	3.13 × 10^3^	2.49 × 10^4^
	Epoxy decomposition, free-radical^b^	1.7	1.7	1.21 × 10^6^	1.29 × 10^7^	1.21 × 10^6^	1.29 × 10^7^
	Epoxy decomposition, in-complex^c^	1.7	1.7	7.19 × 10^5^	9.99 × 10^6^	5.40 × 10^5^	7.50 × 10^6^
	Intermolecular H-shift	3.5	4.0	6.79 × 10^6^	2.96 × 10^6^	6.30 × 10^6^	2.75 × 10^6^

PhC(O)O	β-Scission, free-radical^b^	1.1^d^	1.1^d^	1.97 × 10^7^	2.49 × 10^8^	1.97 × 10^7^	2.49 × 10^8^
	β-Scission, in-complex^c^	1.1	1.0	1.05 × 10^7^	1.32 × 10^8^	6.40 × 10^6^	8.02 × 10^7^

a Only the β-scission reaction from the hydroxymethyl side is reported here (R_m_3.2.1 in Fig. 3, see Section 3.2 for further discussion).

b The epoxy formation and decomposition reactions of PhCH_2_O˙ and the β-scission reaction of PhC(O)O˙ only have one conformer for each species. Thus, for these reactions *k*_MC-TST_ = *k*_LC-TST_.

c High-energy dimers with a small imaginary frequency were excluded from *k* calculations (see Sections 3.3 and 3.4 for detailed discussion).

d The reactant monomer obtained from IRC showed nonphysical imaginary frequencies after DFT optimization. Instead, a reactant monomer with the same geometry (RMSD = 0.09 Å) was used to calculate *κ*_min_ values.

### 3.1 The AceO system

**Fig. 2:**
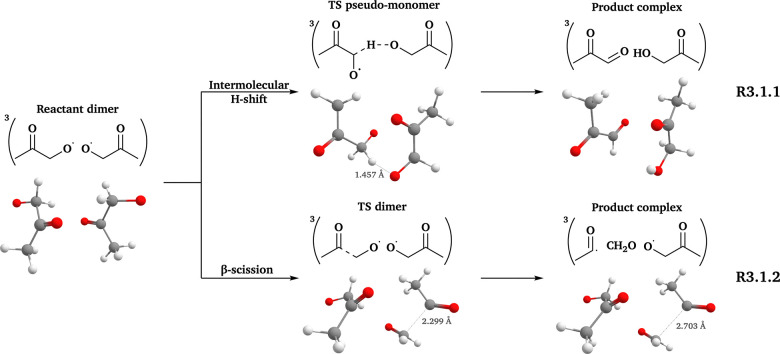
Reaction scheme of ^3^(AceO⋯OAce). All 3D structures presented here are conformers with the lowest *G* after the ωB97X-D3 (Grid3) optimization and filtering. Color coding: gray for C, white for H, and red for O.

Structures of key species during the β-scission and intermolecular H-shift reactions are shown in Fig. 2. Given the relatively simple structure of AceO˙, only a small number of conformers were found for the AceO system after the sampling workflow (see Table 3). Rate coefficients calculated at different levels of theory are listed in Table 4.

Table 4 and previous theoretical studies indicate that the in-complex β-scission is among the fastest of the four reaction channels of ^3^(AceO⋯OAce): its rate coefficient (10^9^–10^10^ s^−1^) exceeds that of the intermolecular H-shift (10^5^–10^7^ s^−1^, this study) and ISC (4 × 10^8^ s^−1^ (ref. 14 and 16)) reactions, and is comparable to that of dissociation (1.5 × 10^9^ s^−1^ (ref. 13)). Moreover, for all four methods employed to compute *k* values, the presence of another AceO˙ in ^3^(AceO⋯OAce) consistently increases the β-scission rate (see the *k* values in the same column of Table 4).

However, in contrast to the high theoretical *k* values, accretion products corresponding to the β-scission reaction (acetonyl acetate, C_5_H_8_O_3_) were only observed at concentrations close to the detection limit in a recent experimental study.^18^ Meanwhile, peroxide products (C_6_H_10_O_4_) corresponding to ISC were more abundant. The discrepancy between theoretical and experimental results implies that competing processes, rather than direct recombination on the triplet surface, may take place after the β-scission reaction. Our preliminary calculations on the ^3^(CH_3_C(O)⋯OAce) complex¶¶This complex corresponds to the product complex of the β-scission reaction after ejecting the formaldehyde. The processes can be summarized as follows: ^3^(AceO⋯OAce) → ^3^(CH_3_C(O)⋯CH_2_O⋯OAce) → ^3^(CH_3_C(O)⋯OAce) + CH_2_O. indicate that although recombination on the triplet surface is thermodynamically feasible, it is prevented by a substantial barrier (see Table S11). Thus, the formation of ROR′-type products likely requires an ISC after β-scission.

For the intermolecular H-shift reaction, our *k*(DFT) values agree well with the reported value (8.85 × 10^6^ s^−1^ (ref. 14)), while our *k*(CC) values are two orders of magnitude higher than the reported value (1.62 × 10^3^ s^−1^ (ref. 14)). The lowest-energy TS H-shift conformer found by our conformer sampling workflow has lower *E*_elzc_ and *G* than that reported by Hasan *et al.*^14^ (see Table S12), suggesting that part of this variation may arise from different geometries used for CCSD(T) calculations. In addition, subtle differences in the CCSD(T) methods employed by the two studies may also contribute.

### 3.2 The β-ISOPO system

**Fig. 3:**
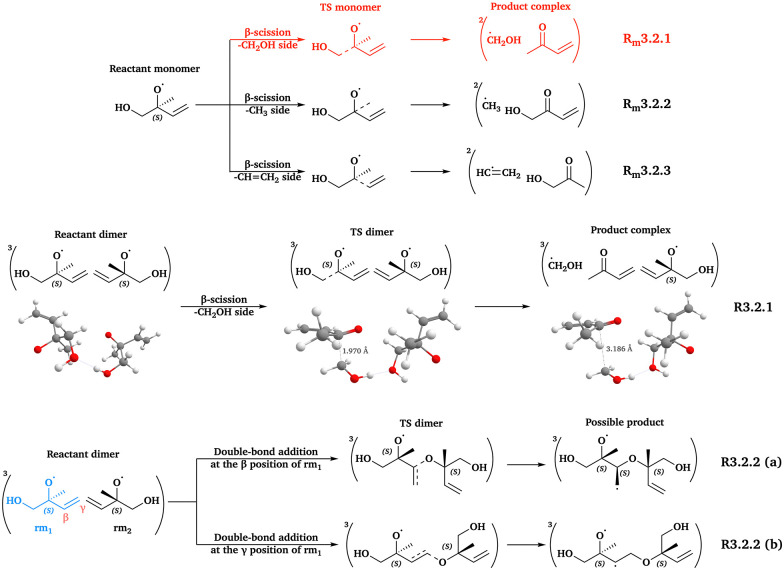
Reaction scheme of the β-ISOPO system. All 3D structures presented are the lowest-*G* conformers, with the same level of theory and color coding as in Fig. 2.

The increased structural complexity of the β-ISOPO system leads to several possible pathways: β-scissions from the hydroxymethyl (R_m_3.2.1 in Fig. 3), methyl (R_m_3.2.2 in Fig. 3) and ethenyl (R_m_3.2.3 in Fig. 3) sides; and radical-double bond addition reactions at the β position (R3.2.2 (a) in Fig. 3) and the γ position (R3.2.2 (b) in Fig. 3). Intermolecular H-shift reactions are unlikely to occur, as no “aldehyde + alcohol” products can be formed for ^3^(β-ISOPO⋯β-ISOPO).

Given the expected large number of reaction channels and conformers of the β-ISOPO system, we first calculated the energy barriers (Δ_b_*E*_elzc_ and Δ_b_*G*, as defined in Table 2) of the following reactions, prior to comprehensive conformer sampling: three β-scission channels of isolated β-ISOPO˙ (R_m_3.2.1–3.2.3) and two radical-double bond addition channels of ^3^(β-ISOPO⋯β-ISOPO) (R3.2.2 (a) and (b)). This step aimed to determine the energetically most favorable pathway, which was then selected for systematic conformer sampling. The barrier heights are listed in Table S13.

As shown in Table S13, R_m_3.2.1 has significantly lower energy barriers than the other channels. The barrier heights of the three β-scission channels align with the trend of unimolecular RO˙ reactions predicted by the structure–activity relationship (SAR) models^19^ (Δ_b_*E*_elzc_(–CH_2_OH) < Δ_b_*E*_elzc_(–CH_3_) < Δ_b_*E*_elzc_(–CH=CH_2_)). Furthermore, differences among Δ_b_*E*_elzc_ and Δ_b_*G* values are large enough that the presence of another β-ISOPO˙ is unlikely to change this conclusion. Thus, the systematic conformer sampling workflow was applied only to β-scission reactions from the hydroxymethyl side (R_m_3.2.1 and R3.2.1 in Fig. 3). The number of conformers and calculated *k* values are presented in Tables 3 and 4, respectively.

For R_m_3.2.1, our *k*(CC) values (2.33 × 10^8^ s^−1^) are around 10 times larger than the reported values (10^7^ s^−1^ at 300 K and 760 Torr).^84,85^ This discrepancy may result from different computational methods and kinetic models between earlier studies and this study (*e.g.* B3LYP^84,85^ and ωB97X-D3, CCSD(T)^84,85^ and DLPNO-CCSD(T)-F12, and RRKM^85^ and elementary TST, where in each pair the latter method is used in this study).

According to the first two sections in Table 4, the presence of another RO˙ in ^3^(RO⋯OR) has a stronger impact on β-scission rates for the β-ISOPO system than for AceO. For β-ISOPO, the *k*(in-complex) values are over two orders of magnitude higher than *k*(free-radical) values. We note that the Δ_b_*G* of R3.2.1 is only 1.30 kcal mol^−1^ at the ωB97X-D3 (Grid3) level, implying that elementary TST may not provide accurate *k* values.^86^ Nevertheless, the notable difference in barrier heights (Δ_b_*G*(free-radical, DFT) − Δ_b_*G*(in-complex, DFT) = 3.34 kcal mol^−1^) supports our qualitative conclusion: the in-complex β-scission reaction of β-ISOPO proceeds much faster than its free-radical counterpart.

### 3.3 The PhCH_2_O system

**Fig. 4:**
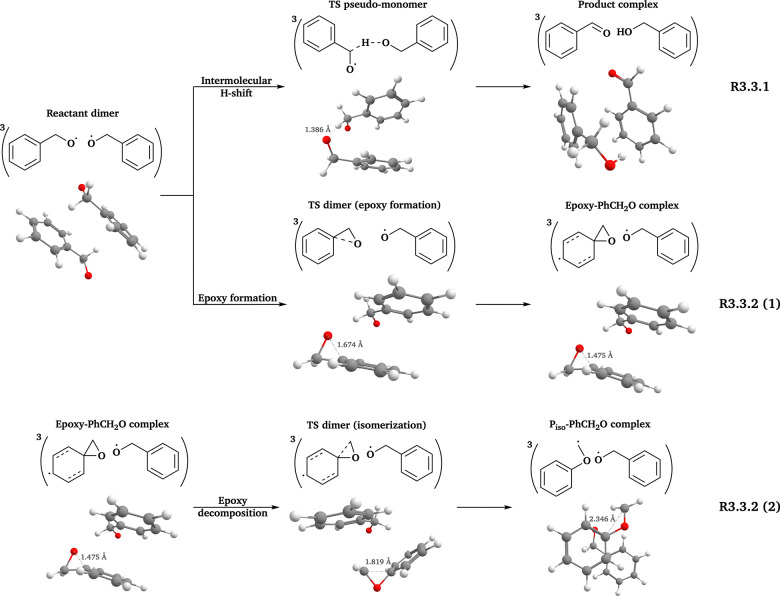
Reaction scheme of the PhCH_2_O system. All 3D structures presented are the lowest-*G* conformers, with the same level of theory and color coding as in Fig. 2.

The β-scission reaction of isolated PhCH_2_O˙ has large energy barriers (Δ_b_*E*_elzc_ = 25.98 kcal mol^−1^ and Δ_b_*G* = 25.35 kcal mol^−1^) at the ωB97X-D3 (Grid3) level. Since the difference in Δ_b_*E* values between in-complex and free-radical reactions is typically less than 5 kcal mol^−1^ (see Section S5 in the SI), the β-scission channel is unlikely to be significant for ^3^(PhCH_2_O⋯OCH_2_Ph) as well. We then examined other possible free-radical reactions of PhCH_2_O˙ and proposed an epoxy formation–decomposition pathway (R3.3.2(1) and (2) in Fig. 4). Similar epoxy formation–decomposition processes have been reported for unsaturated aliphatic RO˙.^22,87^ The conformer sampling workflow mentioned in Section 2 was applied to the species involved in epoxy formation–decomposition and intermolecular H-shift reactions.

Each monomer of the PhCH_2_O system (see Table 3) has only one conformer because of the rigid benzene ring. Thus, *k*(MC-TST) equals *k*(LC-TST) for free-radical epoxy formation and decomposition reactions. Besides, one high-energy TS dimer of the epoxy decomposition reaction showed a second small imaginary frequency (−8.39 cm^−1^), which could not be eliminated by the Grid3 optimization or manual displacements as described in Section 2.3. This conformer was therefore excluded from the *k* calculation.

Table 4 summarizes the *k* values for the free-radical and in-complex reactions of the PhCH_2_O system. Compared to earlier studies,^22,87^ the epoxy formation reaction shows a larger energy barrier in isolated PhCH_2_O˙ (Δ_b_*E*_elzc_(CC, this study) = 12.86 kcal mol^−1^) than in aliphatic RO˙ (Δ_b_*E*(coupled cluster, earlier studies) = 4.60^22^–9.5^87^ kcal mol^−1^). This gap is significant regardless of the different coupled cluster methods applied. We further examined their reaction energies (Δ_r_*E*, defined in Table 2). The epoxy formation reaction in aliphatic RO˙ is isoergic or slightly endoergic (Δ_r_*E*(coupled cluster, earlier studies) = −0.66–2.23 kcal mol^−1^ (ref. 22)), while in PhCH_2_O˙ it is much more endoergic (Δ_r_*E*_elzc_(CC) = 10.27 kcal mol^−1^) and, therefore, thermodynamically less favorable.

Although the epoxy decomposition channel has *k* values comparable to those of intermolecular H-shift, its contribution is not expected to exceed 1% of the final products. This is because its prerequisite step, epoxy formation, proceeds around two orders of magnitude more slowly than the intermolecular H-shift (see Table 4).

Unlike the two systems mentioned in Sections 3.1 and 3.2, the presence of another PhCH_2_O˙ does not exhibit a consistent effect on the reactions of ^3^(PhCH_2_O⋯OCH_2_Ph): it slightly increases the *k* values of epoxy formation, but decreases those of epoxy decomposition (see Table 4).

### 3.4 The PhC(O)O system

**Fig. 5:**
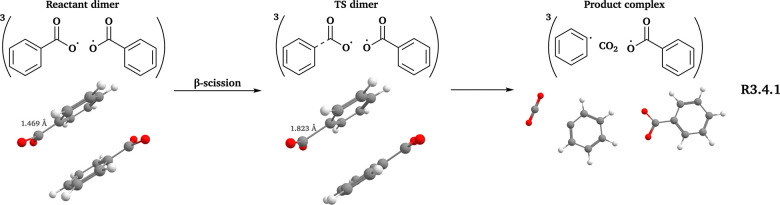
Reaction scheme of the PhC(O)O system. All 3D structures presented are the lowest-*G* conformers, with the same level of theory and color coding as in Fig. 2.

Due to the structural constraints of PhC(O)O˙, only the β-scission channel (see Fig. 5) is feasible for this system, and the number of conformers is relatively small (see Table 3). Similar to the PhCH_2_O system, one high-energy reactant dimer and two high-energy TS dimers of the β-scission in ^3^(PhC(O)O⋯O(O)Ph) showed small imaginary frequencies that could not be eliminated after using the techniques mentioned in Section 2.3. Thus, these three conformers were not included when calculating *k*.

As shown in Table 4, free-radical and in-complex β-scission reactions are relatively fast for the PhC(O)O system. The presence of another PhC(O)O˙ in the complex increases *k*(CC) while decreasing *k*(DFT). However, based on previous studies on CH_3_C(O)O˙,^88,89^ the CO_2_ ejection transition state likely has substantial multireference characters, suggesting that neither *k*(DFT) nor *k*(CC) is particularly accurate.

### 3.5 General findings of the four systems

**Fig. 6:**
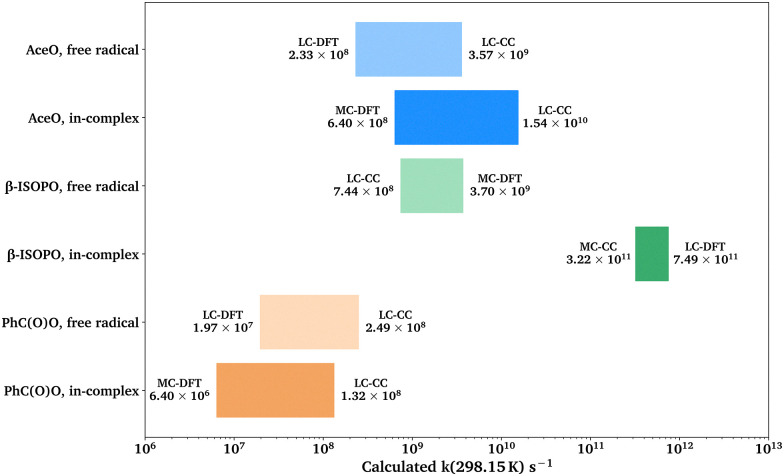
The range of free-radical and in-complex β-scission reactions of different systems. The origin of the *x*-axis is set to 10^6^ s^−1^. Acronyms: MC for MC-TST, LC for LC-TST, DFT for ωB97X-D3 (Grid3), and CC for DLPNO-CCSD(T)-F12.

We plotted the range of *k* for β-scission reactions in AceO, β-ISOPO and PhC(O)O systems, as shown in Fig. 6. The β-scission reaction is kinetically unfeasible for the PhCH_2_O system and is thus not included in the figure.

From Fig. 6, the in-complex β-scission reaction roughly follows the trend *k*(β-ISOPO) > *k*(AceO) > *k*(PhC(O)O), which is consistent with the SAR-predicted order of unimolecular RO˙ reactions.^19,87^ While Fig. 6 shows the overall magnitude of *k* for β-scission reactions, we also evaluate factors that influence the calculated *k* values of all studied reaction channels. These factors include: (1) the reaction takes place in ^3^(RO⋯OR) or in isolated RO˙. (2) *k* was calculated using the MC-TST or LC-TST method. (3) The Δ_b_*G* values were calculated at the ωB97X-D3 (Grid3) or DLPNO-CCSD(T)-F12 level of theory.

To quantify the effects of those factors on *k*, we defined three parameters: *p*(d/m), *p*(MC/LC) and *p*(CC/DFT). For computing *p*(d/m) (eqn (3)) and p(CC/DFT) (eqn (5)), only *k*(LC-TST) values are used to eliminate the effect of multiple conformers. Since intermolecular H-shift reactions involve two RO˙ in the ^3^(RO⋯OR) complex, *p*(d/m) does not apply to them. *p*(MC/LC) reflects the effects of multiple conformers and is computed using only *k*(in-complex) values. For some free-radical reactions, there is only one conformer for each species (see Table 3), and consequently, their *p*(MC/LC) always equals 1. The calculated *p* values are listed in Table 5.3
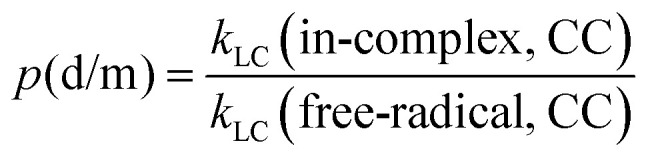
4
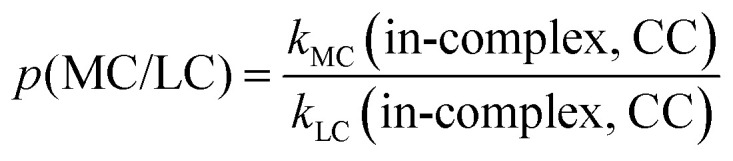
5
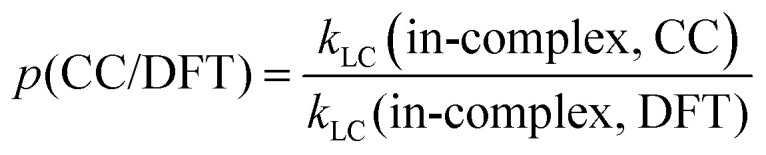


**Table 5:** Results of *p*(d/m), *p*(MC/LC) and *p*(CC/DFT). Parameters were calculated using eqn (3), (4) and (5), respectively. Definitions of *k*(DFT) and *k*(CC) are explained in Table 2

System and reaction	*p*(d/m)	*p*(MC/LC)	*p*(CC/DFT)
AceO, β-scission	4.33	0.39	9.35
AceO, intermolecular H-shift	—	1.44	0.07
β-ISOPO, β-scission	868.81	0.50	0.86
PhCH_2_O, epoxy formation	1.46	1.07	7.96
PhCH_2_O, epoxy decomposition	0.77	0.75	13.89
PhCH_2_O, intermolecular H-shift	—	0.93	0.44
PhC(O)O, β-scission	0.53	0.61	12.52

According to Tables 4 and 5, the formation of ^3^(RO⋯OR) significantly increases the β-scission rates of aliphatic systems. In particular, the presence of the other β-ISOPO˙ raises the *k*(β-scission) by a factor of 868 at the DLPNO-CCSD(T)-F12 level. This increase possibly results from an intermolecular hydrogen bond that stabilizes the TS dimer (O_3_–H_12_⋯O_1_ in Fig. S2b). Although the TS monomer also has a hydrogen bond (O_1_–H_3_⋯O_2_ in Fig. S2a), it is much weaker compared to its counterpart in the TS dimer: the O⋯H distance is longer (2.4 Å *vs.* 1.7 Å in the TS dimer), and the O–H⋯O angle is less favorable (103° *vs.* 177° in the TS dimer).

In addition, the binding energy of ^3^(RO⋯OR) may also affect the *p*(d/m) values. For example, ^3^(β-ISOPO⋯β-ISOPO) is among the strongest-bound complexes (*D*_elzc_(DFT) = 7.96 kcal mol^−1^ and *D*_elzc_(CC) = 6.52 kcal mol^−1^; see Table S2), and its *p*(d/m) is exceptionally large. However, the other three complexes have similar binding energies (within 6–8 kcal mol^−1^, see Table S2), while their *p*(d/m) values are between 0.5 and 5. This observation suggests that while the binding energy may contribute to differences between *k*(in-complex) and *k*(free-radical), it is unlikely to be the determining factor.

*p*(MC/LC) depends on the relative number of low-energy TS and reactant conformers (see Table 3). For example, the intermolecular H-shift reaction of ^3^(AceO⋯OAce) has 11 low-energy TS conformers, leading to *p*(MC/LC) > 1. However, all *p*(MC/LC) values are between 0.3 and 1.5, indicating that multiple conformers change the *k* value by less than a factor of three. Thus, the multi-conformer effects are a relatively minor source of error, as long as the global minimum conformers (with the lowest *G*) have been correctly identified by the sampling workflow.

*p*(CC/DFT) is an indicator of consistency between *k*(DFT) and *k*(CC) values. If the differences between the DFT and CCSD(T) results were consistent and systematic among the four systems, the four *p*(CC/DFT) values could be expected to be similar. However, *p*(CC/DFT) varies from 0.07 to 13.89 (see Table 5). Notably, the two H-shift reactions have *p*(CC/DFT) < 1, while the β-scission reactions have *p*(CC/DFT) values close to or above one. Considering the typical uncertainties when applying DFT and CCSD(T) methods to complex open-shell systems, the disagreement shown in Table 5 is within an acceptable range.

## 4 Conclusions

Based on previous studies,^14,15,31^ we developed a systematic conformer sampling workflow for the key intermediate in RO_2_˙ recombination reactions, ^3^(RO⋯OR). This workflow was applied to four representative alkoxy systems in the atmosphere: AceO, β-ISOPO, PhCH_2_O and PhC(O)O. The rate coefficients for typical reactions were computed at the ωB97X-D3/ma-def2-TZVP (Grid3) and UHF-DLPNO-CCSD(T)-F12/cc-pVDZ-F12 levels of theory.

We examined the established channels of these systems, including β-scission and intermolecular H-shift reactions. Our workflow improved the conformer sampling, especially for intermolecular H-shift reactions. For example, we identified a lower-energy TS dimer for the β-scission of ^3^(AceO⋯OAce), compared to the best TS conformer reported previously.^14^ Furthermore, we investigated two novel pathways: RO˙ addition to the double bond of the other RO˙ in the complex (R3.2.2(a) and (b) in Fig. 3) and epoxy formation–decomposition of PhCH_2_O˙, leading to a phenoxymethyl radical (R3.3.2(1) and (2) in Fig. 4). However, neither novel pathway was competitive compared to in-complex β-scission and intermolecular H-shift reactions (see Table 4).

Our conformer sampling workflow was tested and found to be robust for both aliphatic and aromatic systems. Including multiple conformers in *k* calculations has a modest effect (less than a factor of three, see Table 5), provided that the lowest-energy conformers have been correctly identified. On the other hand, CCSD(T) energy corrections influence *k* values by less than a factor of 15 (see Table 5). Interestingly, our CCSD(T) corrections tend to slow down intermolecular H-shift reactions, while accelerating β-scission reactions (see Table 5).

For β-scission reactions in the other three alkoxy systems, the *k*(in-complex) and *k*(free-radical) values are comparable. However, in the β-ISOPO system, the *k*(in-complex) is two orders of magnitude higher than *k*(free-radical). This rate enhancement is likely due to the intermolecular hydrogen bond that stabilizes the transition state (see Fig. S2).

Our observations on *k* values tentatively suggest that, for β-scission reactions, *k*(free-radical) can constrain the lower limit of *k*(in-complex), as well as estimate the order-of-magnitude for *k*(in-complex) in relatively simple or weakly-bound systems. However, for aerosol-relevant systems with H-bonding functional groups, *k*(in-complex) may be significantly enhanced. This may help explain the substantial ester formation detected in α-pinene ozonolysis.^17^

## Author contributions

Hongye Fraise Zhao: investigation (main), methodology (main), formal analysis, and writing – original draft. Lauri Franzon: investigation (support), methodology (support), supervision (support), and writing – review & editing (support). Severi Juttula: software (support) and writing – review & editing (support). Robert Skog: methodology (support) and writing – review & editing (support). Theo Kurtén: project administration, funding acquisition, resources, supervision (main), and writing – review & editing (main). Nanna Myllys: funding acquisition, supervision (support), and writing – review & editing (support).

## Conflicts of interest

There are no conflicts to declare.

## Supplementary Material

CP-028-D5CP04635A-s001

## Data Availability

Supplementary information (SI) contains data supporting the findings in this article. Supplementary information: detailed ORCA output files (in .out format) and ωB97X-D3 (Grid3)-optimized structures (in the .xyz format) of all conformers, which contain necessary information (*e.g.* frequencies and energy values) for *k* calculations, are published in the Zenodo archive [DOI: https://doi.org/10.5281/zenodo.19210535]. See DOI: https://doi.org/10.1039/d5cp04635a.
