# Supplementary material for: Clustering effects on the reactivity of alkoxy radicals: rate coefficients of 3(RO⋯OR) complexes accounting for multiple conformers
Source: Phys Chem Chem Phys. 2026 Apr 17;28(21):13099–110. doi: 10.1039/d5cp04635a (PMC13173404; doi:10.1039/d5cp04635a)
Supplement: CP-028-D5CP04635A-s001 [file CP-028-D5CP04635A-s001.pdf]

Supplementary Information (SI) for Physical Chemistry Chemical Physics.
This journal is © the Owner Societies 2026

Supplementary Information for

Clustering effects on the reactivity of alkoxy radicals: Rate coefficients of ^{3}(RO···OR) complexes accounting for multiple conformers

Hongye Fraise Zhao[†], Lauri Franzon[†], Severi Juttula[†], Robert Skog[†], Nanna Myllys[†] and Theo Kurtén[†*]

[†]*Department of Chemistry, University of Helsinki, A.I. Virtasen aukio 1 (Chemicum), 00560, Helsinki, Finland.*

[*]*E-mail: theo.kurten@helsinki.fi*

Contents

S1 Notations applied throughout the Supplementary Information (SI) **2**

S2 Binding energies of the four alkoxy complexes in this study **3**

S3 Schematic representation of the conformer sampling workflow **4**

S4 Evaluations on the multireference character of the studied alkoxy systems **5**

S5 Relative energy values involved in this study **8**
S5.1 Relative energy values of key stationary points 8
S5.2 Relative energy values of all conformers 11

S6 Supporting data for the AceO system **17**
S6.1 Energy barriers of post-β-scission processes 17
S6.2 Comparison between the TS H-shift conformers reported in this and previous studies 17

S7 Supporting data for the β-ISOPO system **18**
S7.1 Energy barriers of possible reaction channels 18
S7.2 Hydrogen bonds in isolated β−ISOPO$^{\bullet}$ and in the complex 19

S1 Notations applied throughout the Supplementary Information (SI)

For clarity and succinctness, Table S1 summarizes the simplified notations of the types of conformers, computational methods and energy terms employed throughout the SI, complementing Table 2 in the main text.

Table S1 Simplified notations of conformers, computational methods and parameters in SI.

Notation in SI	Detailed descriptions
Conformer (G_{min})	The conformer with the lowest Gibbs free energy value (G_{min}) within its category, identified by the conformer sampling workflow (see Section 2 in the main text).
Conformer (IRC)	The conformer which corresponds to an endpoint of the intrinsic reaction coordinate (IRC) calculation. IRC calculations were performed on the transition state conformers with the lowest G values.
Reactant monomer or "rm"	An isolated $RO^{\bullet}$ radical.
TS monomer or "tsm"	A transition state conformer of intramolecular $RO^{\bullet}$ reactions.
Reactant dimer, "2rm" or "rd"	A triplet alkoxy complex, $^3(RO\cdots OR)$.
TS H-shift conformer	A transition state conformer of the intermolecular H-shift reaction of $^3(RO\cdots OR)$, which involves both $RO^{\bullet}$ in the complex.
TS dimer or "1rm1ts"	A transition state conformer of other intramolecular reaction channels of $^3(RO\cdots OR)$, which takes place within one $RO^{\bullet}$ of the complex.
Product monomer or "pcm"	A product complex conformer of reactions in an isolated $RO^{\bullet}$, obtained from IRC calculations.
Product dimer or "pcd"	A product complex conformer of reactions in $^3(RO\cdots OR)$, obtained from IRC calculations.
f(DFT)	Values calculated at the ωB97X-D3/ma-def2-TZVP (DefGrid3) level of theory, where f can be an energy term (E) or frequency (ω_{i}). This level of theory is also denoted "ωB97X-D3 (Grid3)" for simplicity.
f(CC)	If not specified, it refers to values calculated at the UHF-DLPNO-CCSD(T)-F12/cc-pVDZ-F12 level of theory, where f can be an energy term, diagnostic (e.g. T_1, %TAE$_e(T)$) or other parameters (e.g. $\langle S^2\rangle$, $\Delta\langle S^2\rangle$%). This level of theory is also denoted "DLPNO-CCSD(T)-F12" for simplicity. For cases specified with "ROHF" or "pure-UHF", see Sections S4 and S5.
E_{el}	Electronic energy
E_{elzc}	Zero-point corrected electronic energy
G	Gibbs free energy
D	Dissociation energy of a complex
$\Delta_b E$	The energy barrier of a reaction ($\Delta_b E = E_{\text{TS}} - E_{\text{Reactant}}$), which reflects the kinetic feasibility of the reaction.
$\Delta_r E$	Reaction energy ($\Delta_r E = E_{\text{Product}} - E_{\text{Reactant}}$), which reflects the thermodynamic feasibility of the reaction.

S2 Binding energies of the four alkoxy complexes in this study

The binding energies (D) of four ^{3}(RO···OR) were calculated using Equation 1, with notations defined in Table S1. The D_{elzc} values at ωB97X-D3 (Grid3) and DLPNO-CCSD(T)-F12 levels are reported in Table S2.

$$D_{\mathrm{elzc}} = 2E_{\mathrm{elzc}}(\text{Reactant monomer } (G_{\mathrm{min}})) - E_{\mathrm{elzc}}(\text{Reactant dimer } (G_{\mathrm{min}})) \tag{1}$$

Table S2 Binding energies of the four ^{3}(RO···OR), reported in kcal mol^{-1}. Notations are defined in Table S1.

^{3}(RO···OR)	D_{elzc}(DFT)	D_{elzc}(CC)
^{3}(AceO···OAce)	6.09	6.21
^{3}(β−ISOPO···β−ISOPO)	7.96	6.52
^{3}($PhCH_2O$···OCH_2Ph)	7.28	6.53
^{3}(PhC(O)O···O(O)CPh)	7.38	6.00

S3 Schematic representation of the conformer sampling workflow

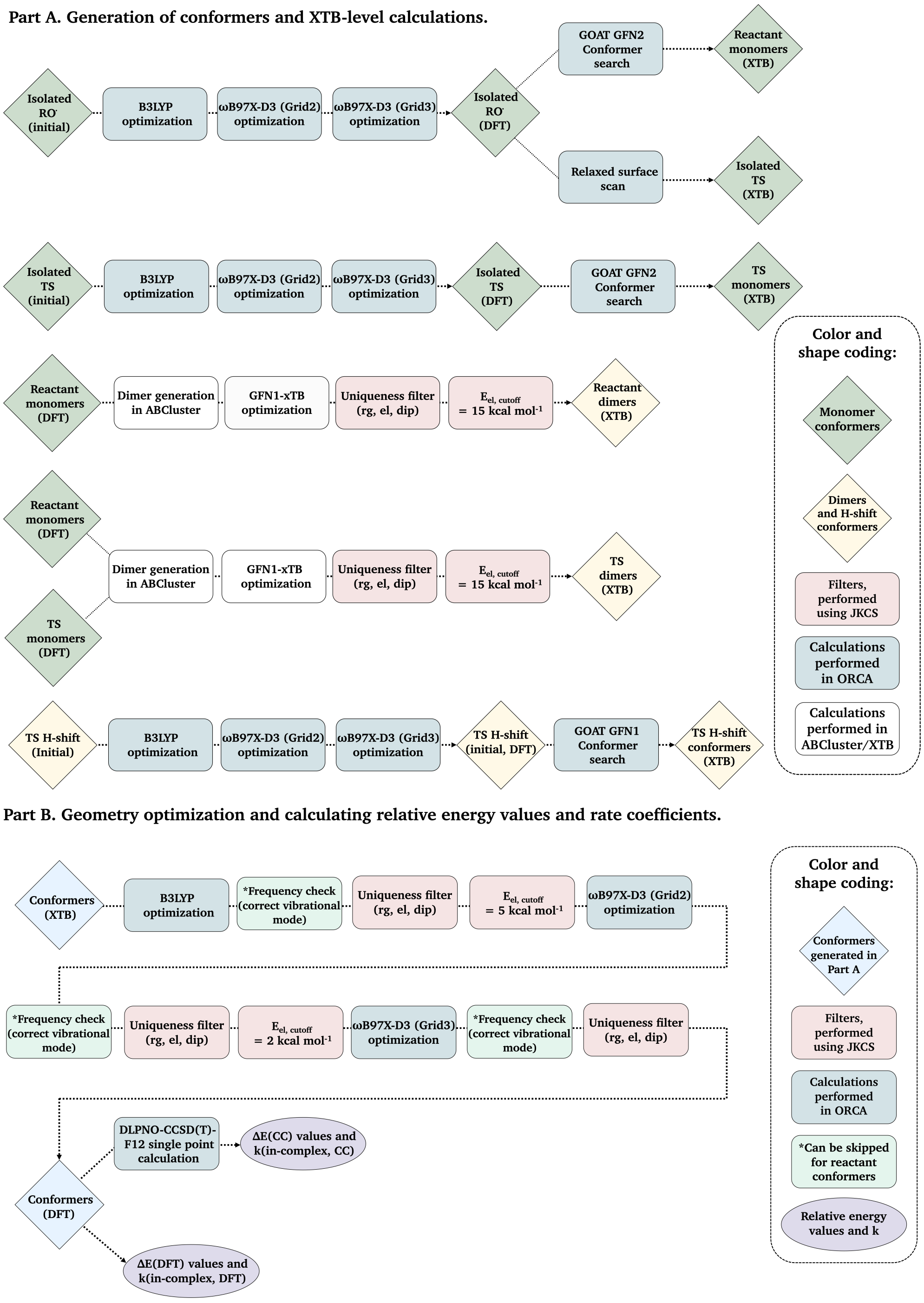

Figure S1 Schematic representation of the conformer sampling workflow. Part A shows the processes including generation the initial structures, conformer search using ABCluster or GOAT, and optimization and filtering at the GFNn-xTB (n=1 or 2) level. Part B shows the optimization and filtering processes after the XTB-level calculations. The color and shape coding panels are listed in the right side of each part.

S4 Evaluations on the multireference character of the studied alkoxy systems

To evaluate the multireference character of the four alkoxy systems in this study, we computed the T_1 and $\%\mathrm{TAE}_e(T)$ diagnostics of significant stationary points, and also examined their spin contamination ($\langle S^2 \rangle$ and $\Delta\langle S^2 \rangle\%$). Those stationary points include the lowest-G TS conformers from the sampling workflow, as well as the IRC endpoints which correspond to the reactants and products of each reaction channel.

In practice, IRC calculations were performed (in both directions) on the lowest-G TS conformers, and the endpoints were then optimized at the ωB97X-D3 (Grid3) level, followed by DLPNO-CCSD(T)-F12 single-point calculations on the optimized structures. The T_1 values were extracted from the coupled-cluster output files. We also computed an energy-based diagnostic, $\%\mathrm{TAE}_e(T)$, which is complementary to the more commonly used T_1.

Conventionally, if a closed-shell organic system has $T_1 < 0.02$, predictions using single-reference methods are expected to be reliable.[1,2] However, previous studies have suggested that relying on the T_1 diagnostic alone may be insufficient for evaluating the multireference character.[3,4] Therefore, we also computed the $\%\mathrm{TAE}_e(T)$ values as a complementary, energy-based diagnostic.

By definition, the total electronic atomization energy (TAE_e) of an organic compound $C_xH_yO_z$ can be calculated by Equation 2, where M stands for the CCSD or CCSD(T) method.[5] The $\%\mathrm{TAE}_e(T)$ diagnostic is then calculated using Equation 3.[6].

$$\mathrm{TAE}_{e,M}(C_xH_yO_z) = xE_{\mathrm{el}}[M](\mathrm{C}) + yE_{\mathrm{el}}[M](\mathrm{H}) + zE_{\mathrm{el}}[M](\mathrm{O}) - E_{\mathrm{el}}[M](C_xH_yO_z) \tag{2}$$

$$\%\mathrm{TAE}_e(T)(C_xH_yO_z) = 100 \times \frac{\mathrm{TAE}_e[\mathrm{CCSD(T)}](C_xH_yO_z) - \mathrm{TAE}_e[\mathrm{CCSD}](C_xH_yO_z)}{\mathrm{TAE}_e[\mathrm{CCSD(T)}](C_xH_yO_z)} \tag{3}$$

Karton et al.[6] have suggested the following criteria for the $\%\mathrm{TAE}_e(T)$ diagnostic: Systems with $\%\mathrm{TAE}_e(T) < 2\%$ are generally dominated by dynamical correlations, which are usually well-described by single-reference methods. On the other hand, systems with $\%\mathrm{TAE}_e(T) > 10\%$ exhibit severe nondynamical correlations, and multireference methods are recommended.

In addition to T_1 and $\%\mathrm{TAE}_e(T)$ diagnostics, we also examined the spin contamination ($\langle S^2 \rangle$) of key conformers, as we employed the unrestricted Hartree-Fock (UHF) type wavefunction as the reference for DLPNO-CCSD(T)-F12 calculations. Spin contamination has been a common problem for UHF treatments, as it may distort the shape of the potential energy surface and, consequently, affect the coupled-cluster energies.[2] Therefore, we extracted the $\langle S^2 \rangle$ values from the output files and computed their deviation percentage from the pure spin state ($\Delta\langle S^2 \rangle\%$) using Equation 4.[7] It is commonly accepted that if $\Delta\langle S^2 \rangle\% < 10\%$, the spin contamination is negligible.[8]

$$\Delta\langle S^2 \rangle\% = \frac{\langle S^2 \rangle - S(S+1)}{S(S+1)} \tag{4}$$

The T_1, %$TAE_e(T)$, $\langle S^2 \rangle$ and $\Delta\langle S^2 \rangle$% values of lowest-$G$ conformers and IRC endpoints in this study are reported in Table S3. For the ωB97X-D3 (Grid3) calculations, the spin contamination is minor ($\Delta\langle S^2 \rangle$%(DFT) < 10%) and thus not reported here.

Table S3 T_1 and %$TAE_e(T)$ diagnostics and spin contamination indicators of key conformers in this study. Parameters were calculated at the DLPNO-CCSD(T)-F12 level of theory. Alkoxy systems and reaction channels are presented in bold.

Conformer Type	T_1	%$TAE_e(T)$	$\langle S^2 \rangle$	$\Delta\langle S^2 \rangle$%
**AceO, β-scission, free radical**				
Reactant monomer[a]	0.0250	1.57	0.76	1.5%
TS monomer (G_{min})	0.0215	1.89	0.88	16.7%
Product monomer (IRC)	0.0188	1.73	0.77	3.2%
**AceO, β-scission, in-complex**[b]				
Reactant dimer (G_{min})	0.0207	1.73	2.09	4.6%
Reactant dimer (IRC)	0.0253	1.63	2.03	1.3%
TS dimer (G_{min})	0.0207	1.73	2.09	4.6%
Product dimer (IRC)	0.0206	1.68	2.04	2.1%
**AceO, Intermolecular H-shift**[b]				
Reactant dimer (IRC)	0.0261	1.65	2.03	1.3%
TS H-shift conformer (G_{min})	0.0283	1.79	2.04	1.9%
Product dimer (IRC)	0.0261	1.76	2.08	4.1%
**β-ISOPO, β-scission, free radical**				
Reactant monomer[a]	0.0162	1.41	0.76	1.8%
TS monomer (G_{min})	0.0211	1.61	0.91	21.9%
Product monomer (IRC)	0.0162	1.46	0.76	1.6%
**β-ISOPO, β-scission, in-complex**				
Reactant dimer (G_{min})	0.0180	1.45	2.05	2.3%
Reactant dimer (IRC)	0.0180	1.45	2.05	2.3%
TS dimer (G_{min})	0.0189	1.54	2.17	8.7%
Product dimer (IRC)	0.0161	1.46	2.09	4.5%
**$PhCH_2O$, Epoxy formation, free radical**				
Reactant monomer (G_{min})	0.0146	1.67	1.14	51.9%
Reactant monomer (IRC)	0.0141	1.68	1.17	56.5%
TS monomer (G_{min})	0.0195	1.90	1.33	77.5%
Isolated epoxy radical[a]	0.0186	1.78	1.18	56.7%
**$PhCH_2O$, Epoxy formation, in-complex**[b]				
Reactant dimer (G_{min})	0.0148	1.70	2.75	37.3%
Reactant dimer (IRC)	0.0147	1.71	2.85	42.3%
TS dimer (G_{min})	0.0173	1.82	2.96	48.1%
Epoxy–$PhCH_2O$ complex (IRC)	0.0171	1.76	2.83	41.4%
**$PhCH_2O$, Epoxy decomposition, free radical**				
Isolated epoxy radical[a]	0.0186	1.78	1.18	56.7%
TS monomer (G_{min})	0.0186	1.87	1.33	78.0%
Product monomer (IRC)	0.0135	1.72	1.14	51.9%
**$PhCH_2O$, Epoxy decomposition, in-complex**				
Epoxy–$PhCH_2O$ complex (G_{min})	0.0170	1.76	2.82	41.2%

Continued on next page

Continued from previous page

Conformer Type	T_1	%TAE$_e(T)$	$\langle S^2 \rangle$	$\Delta\langle S^2 \rangle$%
Epoxy–$PhCH_2O$ complex (IRC)	0.0170	1.75	2.83	41.4%
TS dimer (G_{min})	0.0168	1.80	2.97	48.4%
Product dimer (IRC)	0.0143	1.73	2.78	39.1%
**$PhCH_2O$, Intermolecular H-shift**[c]				
Reactant dimer (IRC)	0.0154	1.68	2.78	38.9%
TS H-shift conformer (G_{min})	0.0181	1.79	2.86	42.9%
Product dimer (IRC, ROHF-UHF)[c]	0.0172	1.76	2.96	47.8%
Product dimer (IRC, pure-UHF)[d]	0.0368	1.88	2.58	28.9%
**PhC(O)O, β-scission, free radical**				
Reactant monomer[e]	0.0364	2.15	1.20	59.5%
TS monomer (G_{min})	0.0167	2.24	1.32	75.4%
Product monomer (IRC)	0.0157	2.01	1.32	76.5%
**PhC(O)O, β-scission, in-complex**				
Reactant dimer (G_{min})	0.0360	2.18	2.85	42.5%
Reactant dimer (IRC)	0.0336	2.13	2.85	42.5%
TS dimer (G_{min})	0.0285	2.23	3.01	50.7%
Product dimer (IRC)	0.0286	2.08	3.01	50.6%

[a] The conformer obtained from IRC has the same geometry as the one identified in the sampling workflow (RMSD < 0.01 Å), and are thus not labeled with brackets.

[b] For reactions labeled "c" within each system, we selected the same "Reactant dimer (G_{min})" was selected as the zero of energy. Parameters of this conformer were reported only once to avoid redundancy.

[c] Results calculated using a UHF-type wavefunction generated from the ROHF-type orbitals, followed by coupled-cluster steps. (See Section S5.1 for detailed discussions.) This procedure is denoted "ROHF-UHF".

[d] Results calculated using a UHF-type wavefunction generated from a pure-UHF initial guess. This procedure is denoted "pure-UHF".

[e] The reactant monomer obtained from IRC showed nonphysical imaginary frequencies after DFT optimization. Instead, a reactant monomer with the same geometry (RMSD = 0.09 Å) was used here.

For the AceO and β-ISOPO systems, some T_1 values exceed the 0.02 threshold, while all %TAE$_e(T)$ values are below 2%. The spin contamination of two TS β-scission monomers are larger than 10%, which is aligned with the feature that spin contamination in general increases as a bond is stretched[2]. In summary, single-reference calculations can be considered reliable for these two systems.

For the $PhCH_2O$ system, most T_1 and %TAE$_e(T)$ values fall below the 0.02 and 2% thresholds, except for the entry denoted "Product dimer (IRC, pure-UHF)" in Table S3. We believe that for the intermolecular H-shift product dimer ($^3(PhCH_2OH \cdots PhCHO)$), the solution obtained by the pure-UHF treatment is an artifact, as discussed in Section S5.1. In addition, some conformers have T_1 and %TAE$_e(T)$ values close to the thresholds, suggesting that nondynamical correlations may play a more important role in the $PhCH_2O$ system compared to typical single-reference systems.

In contrast, the PhC(O)O system is even more affected by nondynamical correlations, especially for the in-complex β-scission reaction, where both T_1 and %TAE$_e(T)$ values are above the thresholds. Therefore, rate coefficients computed at the DLPNO-CCSD(T)-F12 level for this system should be considered as more qualitative than quantitative.

S5 Relative energy values involved in this study

In this section, the relative energy values were calculated using the following equation:

$$\Delta E = E_i - E_{\text{Reactant}(G_{\text{min}})} \tag{5}$$

where i refers to reactant, TS or product conformers; Reactant(G_{min}) refers to the reactant conformer with the lowest G value, which was identified by the conformer sampling workflow; and E represents E_{el}, E_{elzc} or G (see Table S1). All the energy values are reported in kcal mol^{-1}.

S5.1 Relative energy values of key stationary points

As discussed in Section S4, the DLPNO-CCSD(T)-F12 results of all IRC endpoints allows us to estimate the upper limit of reaction (free) energies ($\Delta_r E$, see Table S1), as a more thorough sampling of product conformers would likely decrease the $\Delta_r E$ values rather than increase them.

However, we identified a problem when computing the DLPNO-CCSD(T)-F12 energies of ^{3}($PhCH_2OH \cdots PhCHO$), which corresponds to the product dimer of the intermolecular H-shift reaction in the $PhCH_2O$ system.

Originally, we applied the default settings in ORCA 6.0.1 for the initial guess (i.e. the model potential method[9], as implied in our output files) and a UHF-type wavefunction as the reference for subsequent coupled-cluster calculations. This procedure is denoted "pure-UHF". According to Table S4, when pure-UHF treatment was applied to compute coupled-cluster energies, this conformer lies around 30 kcal mol^{-1} higher than the lowest-G reactant dimer. In contrast, at the same level of theory, the sum of isolated product molecules (^{1}($PhCH_2OH$) and ^{3}(PhCHO)) lies over 14 kcal mol^{-1} below the reactant dimer. This would imply that the product complex is unbound by more than 40 kcal mol^{-1} (see Equation 6 for computing the dissociation energy, D_{elzc}), which is physically not reasonable.

$$^3(\text{PhCH}_2\text{OH}\cdots\text{PhCHO}) \rightarrow {}^1(\text{PhCH}_2\text{OH}) + {}^3(\text{PhCHO}) \quad D_{\text{elzc}}(\text{CC, pure-UHF}) = -44.70 \text{ kcal mol}^{-1} \tag{6}$$

On the other hand, the DFT-level results show an internally consistent pattern, where the product complex lies about 20 kcal mol^{-1} below the reactant dimer, and 7 kcal mol^{-1} below the isolated products. One possible explanation to the discrepancy between DFT and coupled-cluster results is that the UHF calculation has converged on an artificial (local minimum) solution[10], leading to spurious coupled-cluster energies. Moreover, the result employing pure-UHF treatment has the highest T_1 value in this study (T_1 = 0.0368, see Table S3), which supports our hypothesis that its energy is associated with a large error.

As a test, we first computed the orbitals of ^{3}($PhCH_2OH \cdots PhCHO$) using the restricted open-shell Hartree-Fock (ROHF) method. These ROHF-type orbitals were then used as the initial guess to generate a UHF-type wavefunction, which, after SCF convergence, served as the reference for subsequent coupled-cluster steps. This procedure is denoted "ROHF-UHF".

Surprisingly, introducing the ROHF orbitals led to a substantially lower absolute energy after the UHF calculation ($E_{\text{post UHF}}$(ROHF-UHF) = −688.1726 Hartree, $E_{\text{post UHF}}$(only UHF) = −688.0600 Hartree, with a difference

of 70.62 kcal mol^{-1}). As relaxing the restrictions on orbitals, i.e. shifting from ROHF to UHF, should lower the energy values instead of raising them, this observation strongly supports our hypothesis that the pure-UHF solution is an artificial local minimum, which is fortuitously avoided when ROHF orbitals are used to generate the initial guess. Furthermore, relative energies obtained from the ROHF-UHF procedure are internally consistent at the coupled-cluster level: The product complex lies below the reactant dimer, as well as below the isolated products (D_{elzc}(CC, ROHF-UHF) = 6.61 kcal mol^{-1}).

Finally, we note that the T_1 diagnostic of the ROHF-UHF calculation is considerably lower (T_1 = 0.0172, see Table S3) than that of the pure-UHF calculation. This further suggests that the pure-UHF result is an artifact and can be safely discarded. Interestingly, the ROHF-UHF procedure also leads to increased spin contamination compared to the pure-UHF procedure ($\langle S^2 \rangle$(ROHF-UHF) = 2.96, $\langle S^2 \rangle$(pure-UHF) = 2.58, see Table S3).

Table S4 Relative energy values of the intermolecular H-shift reaction in $^3(PhCH_2O\cdots OCH_2Ph)$. Notations are defined in Table S1.

Conformer Type	ΔE_{el}(DFT)	ΔE_{el}(CC)	ΔE_{elzc}(DFT)	ΔE_{elzc}(CC)	ΔG(DFT)	ΔG(CC)
Reactant dimer (G_{min})	0.00	0.00	0.00	0.00	0.00	0.00
Reactant dimer (IRC)	4.62	4.10	3.93	3.41	4.83	4.31
TS H-shift conformer (G_{min})	8.95	9.52	7.40	7.96	8.88	9.44
Product dimer (IRC, pure-UHF)[a]	-18.04	30.53	-17.03	31.54	-16.82	31.75
Product dimer (IRC, ROHF-UHF)[b]	-18.04	-20.77	-17.03	-19.77	-16.82	-19.55
$^1(PhCH_2OH) + {}^3(PhCHO)$[c]	-10.13	-13.47	-9.82	-13.16	-22.03	-25.37

[a] Results calculated using a UHF-type wavefunction generated from a pure-UHF initial guess. This procedure is denoted "pure-UHF".
[b] Results calculated using a UHF-type wavefunction generated from the ROHF-type orbitals, followed by coupled-cluster steps. This procedure is denoted "ROHF-UHF".
[c] Structures of isolated $^1(PhCH_2OH)$ and $^3(PhCHO)$ molecules were obtained from the product dimer and optimized at the ωB97X-D3 (Grid3) level. DLPNO-CCSD(T)-F12 calculations (with the pure-UHF procedure) were performed on top of the optimized structures. Relative energy values reported here are the sum of that of isolated $^1(PhCH_2OH)$ and $^3(PhCHO)$.

The relative energy values of each reaction channel are reported in Table S5. Except for three channels of the $PhCH_2O$ system (free radical and in-complex epoxy formation reactions, which are both kinetically uncompetitive; and the free radical epoxy decomposition reaction, which is constrained by the prerequisite epoxy formation step), the studied reactions are either exothermic or close to thermoneutral. Therefore, thermodynamic feasibility is unlikely to be a major limiting factor for the reactions in this study.

Table S5 Relative energy values of significant stationary points in this study. Notations are defined in Table S1. Alkoxy systems and reaction channels are presented in bold.

Conformer Type	ΔE_{el}(DFT)	ΔE_{el}(CC)	ΔE_{elzc}(DFT)	ΔE_{elzc}(CC)	ΔG(DFT)	ΔG(CC)
**AceO, β-scission, free radical**						
Reactant monomer[a]	0.00	0.00	0.00	0.00	0.00	0.00
TS monomer (G_{min})	7.57	5.96	6.54	4.93	6.06	4.44
Product monomer (IRC)	5.94	2.55	4.12	0.73	1.79	-1.60
**AceO, β-scission, in-complex**[b]						
Reactant dimer (G_{min})	0.00	0.00	0.00	0.00	0.00	0.00
Reactant dimer (IRC)	1.60	1.98	1.64	2.03	1.63	2.02

Continued on next page

Continued from previous page

Conformer Type	ΔE_{el}(DFT)	ΔE_{el}(CC)	ΔE_{elzc}(DFT)	ΔE_{elzc}(CC)	ΔG(DFT)	ΔG(CC)
TS dimer (G_{min})	6.23	4.91	5.04	3.72	4.88	3.56
Product dimer (IRC)	5.99	3.52	4.40	1.94	3.14	0.67
**AceO, Intermolecular H-shift**[b]						
Reactant dimer (IRC)	3.10	3.34	3.02	3.26	3.52	3.76
TS H-shift conformer (G_{min})	8.37	9.96	6.87	8.46	8.28	9.86
Product dimer (IRC)	-27.89	-26.98	-26.22	-25.31	-25.54	-24.63
**β-ISOPO, β-scission, free radical**						
Reactant monomer[a]	0.00	0.00	0.00	0.00	0.00	0.00
TS monomer (G_{min})	6.25	7.06	4.87	5.68	4.64	5.45
Product monomer (IRC)	-5.49	-5.13	-8.00	-7.65	-10.14	-9.79
**β-ISOPO, β-scission, in-complex**						
Reactant dimer (G_{min})	0.00	0.00	0.00	0.00	0.00	0.00
Reactant dimer (IRC)	0.01	0.00	0.00	-0.02	0.00	-0.02
TS dimer (G_{min})	2.05	2.14	1.21	1.30	1.30	1.39
Product dimer (IRC)	-3.81	-4.18	-6.47	-6.83	-8.60	-8.96
**$PhCH_2O$, Epoxy formation, free radical**						
Reactant monomer (G_{min})	0.00	0.00	0.00	0.00	0.00	0.00
Reactant monomer (IRC)	1.69	0.99	2.64	1.93	2.64	1.93
TS monomer (G_{min})	12.99	11.43	12.86	11.30	13.43	11.87
Isolated epoxy radical[a]	10.35	9.95	10.67	10.27	11.23	10.83
**$PhCH_2O$, Epoxy formation, in-complex**[b]						
Reactant dimer (G_{min})	0.00	0.00	0.00	0.00	0.00	0.00
Reactant dimer (IRC)	2.67	2.31	3.11	2.75	3.14	2.78
TS dimer (G_{min})	13.19	11.78	12.56	11.15	12.85	11.44
Epoxy–$PhCH_2O$ complex (IRC)	11.64	11.21	11.46	11.02	11.92	11.48
**$PhCH_2O$, Epoxy decomposition, free radical**						
Isolated epoxy radical[a]	0.00	0.00	0.00	0.00	0.00	0.00
TS monomer (G_{min})	10.79	9.39	9.54	8.13	9.48	8.07
Product monomer (IRC)	-10.79	-11.11	-10.86	-11.17	-11.23	-11.55
**$PhCH_2O$, Epoxy decomposition, in-complex**						
Epoxy–$PhCH_2O$ complex (G_{min})	0.00	0.00	0.00	0.00	0.00	0.00
Epoxy–$PhCH_2O$ complex (IRC)	0.72	0.69	0.73	0.70	0.36	0.34
TS dimer (G_{min})	10.88	9.32	9.72	8.16	9.77	8.21
Product dimer (IRC)	-10.83	-11.06	-10.57	-10.80	-10.62	-10.85
**$PhCH_2O$, Intermolecular H-shift**[b]						
Reactant dimer (IRC)	4.62	4.10	3.93	3.41	4.83	4.31
TS H-shift conformer (G_{min})	8.95	9.52	7.40	7.96	8.88	9.44
Product dimer (IRC, ROHF-UHF)[c]	-18.04	-20.77	-17.03	-19.77	-16.82	-19.55
**PhC(O)O, β-scission, free radical**						
Reactant monomer[d]	0.00	0.00	0.00	0.00	0.00	0.00
TS monomer (G_{min})	9.35	7.85	8.03	6.53	7.53	6.03
Product monomer (IRC)	-1.69	-5.04	-2.89	-6.23	-5.21	-8.55
**PhC(O)O, β-scission, in-complex**						
Reactant dimer (G_{min})	0.00	0.00	0.00	0.00	0.00	0.00

Continued on next page

Continued from previous page

Conformer Type	ΔE_{el}(DFT)	ΔE_{el}(CC)	ΔE_{elzc}(DFT)	ΔE_{elzc}(CC)	ΔG(DFT)	ΔG(CC)
Reactant dimer (IRC)	3.79	1.84	3.55	1.61	3.54	1.60
TS dimer (G_{min})	9.62	8.12	8.37	6.87	7.90	6.40
Product dimer (IRC)	3.98	-0.88	2.55	-2.31	0.65	-4.21

[a] The conformer obtained from IRC has the same geometry as the one identified in the sampling workflow (RMSD < 0.01 Å), and are thus not labeled with brackets.

[b] For reactions labeled "c" within each system, we selected the same "Reactant dimer (G_{min})" was selected as the zero of energy. Energy values of this conformer were reported only once to avoid redundancy.

[c] Results calculated using a UHF-type wavefunction generated from the ROHF-type orbitals, followed by coupled-cluster steps. Results calculated from the "pure-UHF" procedure are listed in Table S4.

[d] The reactant monomer obtained from IRC showed nonphysical imaginary frequencies after DFT optimization. Instead, a reactant monomer with the same geometry (RMSD = 0.09 Å) was used here.

S5.2 Relative energy values of all conformers

For conformers in the four studied alkoxy systems, their relative energy values at ωB97X-D3 (Grid3) ("DFT") and DLPNO-CCSD(T)-F12 ("CC") levels are reported in Tables S6 — S10. The imaginary frequencies of TS conformers calculated at the DFT level (ω_i(DFT)) are also reported in cm^{-1}.

Table S6 Relative energy values and imaginary frequencies of the conformers of the AceO system. Notations are defined in Table S1.

Conformer Name[a]	ΔE_{el}(DFT)	ΔE_{el}(CC)	ΔE_{elzc}(DFT)	ΔE_{elzc}(CC)	ΔG(DFT)	ΔG(CC)	ω_i(DFT)
rm_goatgfn2_02	0.00	0.00	0.00	0.00	0.00	0.00	—
rm_goatgfn2_03	3.42	3.42	3.51	3.51	3.54	3.54	—
tsm_goatgfn2_01	7.57	5.96	6.54	4.93	6.06	4.44	-183.22
tsm_goatgfn2_04	11.70	10.09	10.61	9.00	10.19	8.57	-230.41
pcm_IRC_sci	5.94	2.55	4.12	0.73	1.79	-1.60	—
2rm-2_24_65	0.00	0.00	0.00	0.00	0.00	0.00	—
2rm-2_22_82	0.02	0.02	0.02	0.02	0.03	0.03	—
2rm-2_24_62	-0.03	-0.03	-0.04	-0.04	0.07	0.07	—
2rm-2_17_87	1.48	1.48	1.54	1.54	1.86	1.86	—
2rm-2_16_85	1.50	1.50	1.60	1.60	1.98	1.98	—
2rm-2_8_81	2.35	2.35	2.28	2.28	2.17	2.17	—
2rm-2_1_65	2.86	2.86	2.85	2.85	3.33	3.33	—
1rm1tsm-11_31_51	6.23	4.94	5.04	3.75	4.88	3.59	-94.45
1rm1tsm-11_11_81	7.32	6.03	6.14	4.85	6.14	4.84	-66.57
1rm1tsm-11_29_95	8.03	6.74	7.01	5.72	7.17	5.87	-157.84
rd_IRC_sci	1.60	1.98	1.64	2.03	1.63	2.02	—
pcd_IRC_sci	5.99	3.52	4.40	1.94	3.14	0.67	—
ts_goatgfn1_24	8.37	9.96	6.87	8.46	8.28	9.86	-727.37
ts_goatgfn1_06	8.82	10.41	7.09	8.68	8.28	9.87	-1147.57
ts_goatgfn1_34	9.00	10.59	7.10	8.69	8.48	10.07	-981.69
ts_goatgfn1_15	8.68	10.27	7.25	8.84	8.73	10.32	-934.63

Continued on next page

Continued from previous page

Conformer Name[a]	ΔE_{el}(DFT)	ΔE_{el}(CC)	ΔE_{elzc}(DFT)	ΔE_{elzc}(CC)	ΔG(DFT)	ΔG(CC)	ω_i(DFT)
ts_goatgfn1_10	9.34	10.93	7.56	9.15	8.87	10.46	-1139.15
ts_goatgfn1_31	9.32	10.91	7.64	9.23	8.95	10.54	-694.61
ts_goatgfn1_19	10.91	12.50	8.85	10.44	9.69	11.28	-1158.33
ts_goatgfn1_14	11.06	12.64	8.98	10.57	9.78	11.37	-1180.19
ts_goatgfn1_23	10.75	12.34	8.73	10.32	9.79	11.38	-1468.78
ts_goatgfn1_29	10.67	12.26	8.83	10.42	9.79	11.38	-983.48
ts_goatgfn1_13	10.94	12.53	8.93	10.52	9.87	11.46	-1276.04
rd_IRC_Hshift	3.10	3.34	3.02	3.26	3.52	3.76	—
pcd_IRC_Hshift	-27.89	-26.98	-26.22	-25.31	-25.54	-24.63	—

[a] Conformer names and their corresponding types are defined in Table S1. Additional labels include: "tsm" – TS monomer of the β-scission reaction, "1rm1tsm" – TS dimer of the β-scission reaction, "ts_goatgfn1" – TS conformer of the intermolecular H-shift reaction, "sci" – conformer of the β-scission reaction, "H-shift" – conformer of the intermolecular H-shift reaction.

Table S7 Relative energy values and imaginary frequencies of the conformers of the β-ISOPO system. Notations are defined in Table S1.

Conformer Name[a]	ΔE_{el}(DFT)	ΔE_{el}(CC)	ΔE_{elzc}(DFT)	ΔE_{elzc}(CC)	ΔG(DFT)	ΔG(CC)	ω_i(DFT)
rm_goatgfn2_02	0.00	0.00	0.00	0.00	0.00	0.00	—
rm_goatgfn2_03	0.74	0.74	0.88	0.88	0.88	0.88	—
rm_goatgfn2_06	0.95	0.95	1.02	1.02	1.15	1.15	—
rm_goatgfn2_04	1.23	1.23	1.15	1.15	1.16	1.16	—
rm_goatgfn2_01	1.61	1.61	1.37	1.37	1.29	1.29	—
rm_goatgfn2_05	1.00	1.00	1.20	1.20	1.32	1.32	—
rm_goatgfn2_07	1.77	1.77	1.70	1.70	1.72	1.72	—
rm_goatgfn2_14	2.28	2.28	2.13	2.13	1.94	1.94	—
tsmOH_goatgfn2_03	6.25	7.06	4.87	5.68	4.64	5.45	-401.47
tsmOH_goatgfn2_04	6.69	7.50	5.35	6.15	5.14	5.94	-403.95
tsmOH_goatgfn2_01	6.98	7.79	5.53	6.34	5.33	6.14	-402.79
tsmOH_goatgfn2_05	6.68	7.49	5.40	6.21	5.37	6.18	-337.59
tsmOH_goatgfn2_02	7.13	7.94	5.74	6.55	5.55	6.35	-403.73
tsmOH_goatgfn2_06	7.67	8.47	6.35	7.16	6.21	7.01	-338.09
pcm_IRC_sciOH	-5.49	-5.13	-8.00	-7.65	-10.14	-9.79	—
2rm-2_255_2	0.00	0.00	0.00	0.00	0.00	0.00	—
2rm-2_331_9	0.41	0.41	0.57	0.57	0.83	0.83	—
2rm-2_320_6	1.02	1.02	0.98	0.98	1.01	1.01	—
2rm-2_220_9	1.44	1.44	1.27	1.27	1.12	1.12	—
2rm-2_215_6	1.44	1.44	1.30	1.30	1.17	1.17	—
2rm-2_214_1	1.04	1.04	1.07	1.07	1.18	1.18	—
2rm-2_231_5	1.12	1.12	1.16	1.16	1.18	1.18	—
2rm-2_231_8	1.12	1.12	1.16	1.16	1.18	1.18	—
2rm-2_211_4	1.75	1.75	1.72	1.72	1.47	1.47	—
2rm-2_217_3	1.75	1.75	1.75	1.75	1.51	1.51	—
2rm-2_156_4	2.17	2.17	2.12	2.12	1.89	1.89	—

Continued on next page

Continued from previous page

Conformer Name[a]	ΔE_{el}(DFT)	ΔE_{el}(CC)	ΔE_{elzc}(DFT)	ΔE_{elzc}(CC)	ΔG(DFT)	ΔG(CC)	ω_i(DFT)
2rm-2_163_3	2.06	2.06	2.13	2.13	2.04	2.04	—
2rm-2_166_9	2.06	2.06	2.14	2.14	2.05	2.05	—
2rm-2_80_7	2.18	2.18	1.97	1.97	2.09	2.09	—
2rm-2_296_7	2.18	2.18	1.98	1.98	2.11	2.11	—
2rm-2_291_0	2.18	2.18	2.00	2.00	2.16	2.16	—
2rm-2_41_5	2.13	2.13	2.09	2.09	2.26	2.26	—
2rm-2_219_6	3.21	3.21	2.95	2.95	2.32	2.32	—
2rm-2_167_6	2.57	2.57	2.60	2.60	2.32	2.32	—
2rm-2_163_8	2.58	2.58	2.60	2.60	2.34	2.34	—
2rm-2_215_9	3.21	3.21	2.98	2.98	2.38	2.38	—
2rm-2_63_7	3.23	3.23	2.76	2.76	2.51	2.51	—
2rm-2_61_5	3.23	3.23	2.79	2.79	2.56	2.56	—
2rm-2_212_9	2.99	2.99	2.92	2.92	2.64	2.64	—
2rm-2_24_7	2.78	2.78	2.65	2.65	2.77	2.77	—
1rm1tsm-11_384_6	2.05	2.14	1.21	1.30	1.30	1.39	-286.31
1rm1tsm-11_239_9	2.75	2.84	1.85	1.94	2.14	2.22	-271.08
1rm1tsm-11_373_8	5.28	5.37	4.13	4.21	4.04	4.13	-293.10
1rm1tsm-11_380_4	5.28	5.37	4.13	4.21	4.06	4.14	-292.58
1rm1tsm-11_14_3	5.49	5.57	4.25	4.34	4.27	4.36	-246.59
1rm1tsm-11_363_6	5.84	5.93	4.70	4.79	4.37	4.46	-284.13
1rm1tsm-11_361_9	5.84	5.93	4.73	4.82	4.42	4.50	-283.47
1rm1tsm-11_74_6	6.45	6.54	5.16	5.24	4.86	4.94	-262.54
1rm1tsm-11_135_4	6.05	6.14	4.87	4.96	4.88	4.97	-259.95
1rm1tsm-11_74_3	6.45	6.54	5.18	5.27	4.91	4.99	-262.88
1rm1tsm-11_424_8	6.17	6.26	4.90	4.99	4.99	5.08	-273.05
1rm1tsm-11_71_6	6.73	6.82	5.39	5.47	5.06	5.15	-258.10
1rm1tsm-11_431_5	6.22	6.31	4.96	5.05	5.06	5.15	-284.31
1rm1tsm-11_437_8	6.23	6.32	5.00	5.08	5.09	5.18	-284.95
1rm1tsm-11_309_9	6.66	6.75	5.53	5.62	5.13	5.22	-282.30
1rm1tsm-11_310_7	5.71	5.79	4.94	5.03	5.16	5.24	-286.39
1rm1tsm-11_301_3	6.66	6.74	5.57	5.65	5.18	5.26	-281.86
1rm1tsm-11_304_3	5.71	5.80	4.97	5.06	5.18	5.27	-286.31
1rm1tsm-11_302_9	6.65	6.73	5.56	5.64	5.19	5.28	-282.61
1rm1tsm-11_1_5	6.79	6.87	5.38	5.47	5.21	5.30	-247.72
1rm1tsm-11_311_7	6.23	6.32	5.16	5.25	5.22	5.31	-293.02
1rm1tsm-11_10_6	5.99	6.08	5.01	5.10	5.43	5.52	-243.03
1rm1tsm-11_67_6	7.47	7.55	6.10	6.19	5.68	5.77	-254.25
1rm1tsm-11_4_4	7.64	7.73	6.29	6.38	5.76	5.84	-247.52
1rm1tsm-11_61_9	7.25	7.33	6.08	6.16	6.05	6.14	-257.86
1rm1tsm-11_1_7	7.78	7.87	6.40	6.49	6.06	6.15	-259.16
rd_IRC_sciOH	0.01	0.00	0.00	-0.02	0.00	-0.02	—
pcd_IRC_sciOH	-3.81	-4.18	-6.47	-6.83	-8.60	-8.96	—

[a] Conformer names and their corresponding types are defined in Table S1. Additional labels include: "tsOH" – TS monomer of the β-scission reaction from the hydroxymethyl side, "1rm1tsm" – TS dimer of the β-scission reaction from the hydroxymethyl side, "sciOH" – conformers of the β-scission reaction from the hydroxymethyl side.

Table S8 Relative energy values and imaginary frequencies of the conformers involved in epoxy formation and intermolecular H-shift reactions of the $PhCH_2O$ system. Notations are defined in Table S1.

Conformer Name[a]	ΔE_{el}(DFT)	ΔE_{el}(CC)	ΔE_{elzc}(DFT)	ΔE_{elzc}(CC)	ΔG(DFT)	ΔG(CC)	ω_{i}(DFT)
rm_Gmin	0.00	0.00	0.00	0.00	0.00	0.00	—
rm_IRC_EPO	1.69	0.99	2.64	1.93	2.64	1.93	—
tsmEPO	12.99	11.43	12.86	11.30	13.43	11.87	-535.42
epom[b]	10.35	9.95	10.67	10.27	11.23	10.83	—
2rm-2_8_83	0.00	0.00	0.00	0.00	0.00	0.00	—
2rm-2_10_87	-0.14	-0.14	-0.22	-0.22	0.17	0.17	—
2rm-2_7_79	-0.14	-0.14	-0.20	-0.20	0.21	0.21	—
1rm1tsepo-11_1_41	13.19	11.75	12.56	11.13	12.85	11.42	-489.73
1rm1tsepo-11_3_45	13.20	11.76	12.60	11.16	13.20	11.76	-490.92
1rm1tsepo-11_1_89	13.18	11.74	12.57	11.13	13.32	11.88	-489.52
1rm1tsepo-11_1_58	13.19	11.75	12.59	11.15	13.36	11.92	-489.71
1rm1tsepo-11_1_29	14.07	12.63	13.72	12.28	14.27	12.84	-503.71
1rm1tsepo-11_9_76	14.16	12.73	13.85	12.41	14.56	13.12	-507.49
1rm1tsepo-11_6_77	14.16	12.72	13.86	12.42	14.61	13.17	-507.95
rd_IRC_EPO	2.67	2.31	3.11	2.75	3.14	2.78	—
eporm_IRC_EPO	11.64	11.21	11.46	11.02	11.92	11.48	—
ts_goatgfn1_01	8.95	9.52	7.40	7.96	8.88	9.44	-1198.18
ts_goatgfn1_02	9.28	9.85	7.57	8.14	9.01	9.58	-1234.69
ts_goatgfn1_04	10.03	10.59	8.37	8.94	9.30	9.87	-957.56
rd_IRC_Hshift	4.62	4.10	3.93	3.41	4.83	4.31	-9.59, -7.18[c]
pcd_IRC_Hshift[d]	-18.04	-20.77	-17.03	-19.77	-16.82	-19.55	—

[a] Conformer names and their corresponding types are defined as follows: "rm" – reactant monomer (isolated $PhCH_2O^{\bullet}$), "tsmEPO" – TS monomer of the epoxy formation reaction, "2rm" – reactant dimer ($^3(PhCH_2O\cdots OCH_2Ph)$), "1rm1tsepo" – TS dimer of the epoxy formation reaction, "IRC_EPO" – conformer obtained from IRC calculations of epoxy formation TS conformers, "ts_goatgfn1" – TS conformer of the intermolecular H-shift reaction.

[b] The isolated epoxy radical obtained from IRC has the same geometry as the one identified in the sampling workflow (RMSD < 0.01 Å).

[c] The imaginary frequencies were likely to be caused by numerical noise, which should not influence the tunneling coefficient and relative energy calculations significantly.

[d] Results calculated using a UHF-type wavefunction generated from the ROHF-type orbitals, followed by coupled-cluster steps. Results calculated from the "pure-UHF" procedure are listed in Table S4.

Table S9 Relative energy values and imaginary frequencies of the conformers involved in the epoxy decomposition reaction of the $PhCH_2O$ system. Notations are defined in Table S1.

Conformer Name[a]	ΔE_{el}(DFT)	ΔE_{el}(CC)	ΔE_{elzc}(DFT)	ΔE_{elzc}(CC)	ΔG(DFT)	ΔG(CC)	ω_{i}(DFT)
epom[b]	0.00	0.00	0.00	0.00	0.00	0.00	—
tsmISO	10.79	9.39	9.54	8.13	9.48	8.07	-712.20
pcm_IRC_ISO	-10.79	-11.11	-10.86	-11.17	-11.23	-11.55	—
1epo1rm-11_1_50	0.00	0.00	0.00	0.00	0.00	0.00	—
1epo1rm-11_6_61	0.00	0.00	-0.01	-0.01	0.05	0.05	—
1epo1rm-11_10_85	0.00	0.00	0.03	0.03	0.11	0.11	—

Continued on next page

Continued from previous page

Conformer Name[a]	ΔE_{el}(DFT)	ΔE_{el}(CC)	ΔE_{elzc}(DFT)	ΔE_{elzc}(CC)	ΔG(DFT)	ΔG(CC)	ω_i(DFT)
1epo1rm-11_4_69	0.00	0.00	0.02	0.02	0.15	0.15	—
1epo1rm-11_10_39	0.07	0.07	0.19	0.19	0.20	0.20	—
1epo1rm-11_5_40	0.06	0.06	0.20	0.20	0.26	0.26	—
1epo1rm-11_6_20	0.06	0.06	0.18	0.18	0.26	0.26	—
1epo1rm-11_1_37	0.50	0.50	0.77	0.77	0.76	0.76	—
1epo1rm-11_3_97	0.51	0.51	0.80	0.80	0.79	0.79	—
1epo1rm-11_10_29	0.64	0.64	0.97	0.97	1.10	1.10	—
1epo1rm-11_5_44	0.71	0.71	0.97	0.97	1.12	1.12	—
1epo1rm-11_5_38	0.64	0.64	0.98	0.98	1.13	1.13	—
1rm1tsiso-11_4_75	10.88	9.32	9.72	8.16	9.77	8.21	-698.03
1rm1tsiso-11_1_99	10.89	9.33	9.85	8.29	9.81	8.25	-700.48
1rm1tsiso-11_6_83	10.89	9.33	9.90	8.34	10.01	8.45	-700.67
1rm1tsiso-11_4_55	11.22	9.66	10.12	8.56	10.06	8.50	-701.29
1rm1tsiso-11_2_37	11.55	9.99	10.38	8.82	10.28	8.72	-707.34
1rm1tsiso-11_3_92	11.90	10.34	10.73	9.17	10.51	8.95	-710.61
1rm1tsiso-11_1_92	11.83	10.27	10.65	9.08	10.61	9.05	-714.89
1rm1tsiso-11_8_74	11.96	10.40	10.93	9.36	10.80	9.24	-713.74
1rm1tsiso-11_4_88	11.91	10.35	10.87	9.31	10.82	9.26	-713.82
1rm1tsiso-11_8_92	11.90	10.34	10.88	9.32	10.87	9.31	-713.10
1rm1tsiso-11_9_97	11.91	10.35	10.89	9.33	10.88	9.32	-712.63
1rm1tsiso-11_1_11	12.08	10.52	11.11	9.55	11.26	9.70	-709.12
eporm_IRC_ISO	0.72	0.69	0.73	0.70	0.36	0.34	—
pcd_IRC_ISO	-10.83	-11.06	-10.57	-10.80	-10.62	-10.85	—

[a] Conformer names and their corresponding types are defined as follows: "epom" – isolated epoxy radical, "tsmISO" – TS monomer of the epoxy decomposition reaction, "1epo1rm" or "eporm" –^{3}(Epoxy$\cdots$$OCH_2$Ph) complex, "1rm1tsiso" – TS dimer of the epoxy decomposition reaction, "IRC_ISO" – conformer obtained from IRC calculations of epoxy decomposition TS conformers.

[b] The isolated epoxy radical obtained from IRC has the same geometry as the one identified in the sampling workflow (RMSD < 0.01 Å).

Table S10 Relative energy values and imaginary frequencies of the conformers of the PhC(O)O system. Notations are defined in Table S1.

Conformer Name[a]	ΔE_{el}(DFT)	ΔE_{el}(CC)	ΔE_{elzc}(DFT)	ΔE_{elzc}(CC)	ΔG(DFT)	ΔG(CC)	ω_i(DFT)
rm[b]	0.00	0.00	0.00	0.00	0.00	0.00	—
tsm	9.35	7.85	8.03	6.53	7.53	6.03	-217.81
pcm_IRC_sci	-1.69	-5.04	-2.89	-6.23	-5.21	-8.55	—
2rm-2_9_47	0.00	0.00	0.00	0.00	0.00	0.00	—
2rm-2_8_90	-0.01	-0.01	0.00	0.00	0.17	0.17	—
2rm-2_8_5	0.00	0.00	0.01	0.01	0.20	0.20	—
2rm-2_3_8	0.01	0.01	0.01	0.01	0.22	0.22	—
2rm-2_10_15	0.03	0.03	0.05	0.05	0.26	0.26	—
2rm-2_10_78	0.00	0.00	0.04	0.04	0.30	0.30	—
2rm-2_10_3	0.02	0.02	0.04	0.04	0.34	0.34	—

Continued on next page

Continued from previous page

Conformer Name[a]	ΔE_{el}(DFT)	ΔE_{el}(CC)	ΔE_{elzc}(DFT)	ΔE_{elzc}(CC)	ΔG(DFT)	ΔG(CC)	ω_i(DFT)
2rm-2_4_16	0.03	0.03	0.05	0.05	0.37	0.37	—
2rm-2_1_67	0.15	0.15	0.20	0.20	0.53	0.53	—
1rm1tsm-11_5_92	9.62	8.12	8.37	6.87	7.90	6.40	-215.23
1rm1tsm-11_3_96	9.60	8.10	8.35	6.86	8.09	6.59	-216.28
1rm1tsm-11_4_45	9.68	8.18	8.44	6.94	8.36	6.87	-226.55
1rm1tsm-11_7_88	9.64	8.14	8.42	6.92	8.38	6.89	-220.43
1rm1tsm-11_10_56	9.66	8.16	8.41	6.91	8.49	6.99	-221.92
1rm1tsm-11_3_6	9.64	8.15	8.44	6.94	8.58	7.08	-223.72
1rm1tsm-11_1_25	9.70	8.20	8.46	6.96	8.67	7.17	-227.37
rd_IRC_sci	3.79	1.84	3.55	1.61	3.54	1.60	-6.78[c]
pcd_IRC_sci	3.98	-0.88	2.55	-2.31	0.65	-4.21	-10.48, -9.36[c]

[a] Conformer names and their corresponding types are defined in Table S1. Additional labels include: "tsm" – TS monomer of the β-scission reaction, "1rm1tsm" – TS dimer of the β-scission reaction, "sci" – conformer of the β-scission reaction.

[b] The reactant monomer obtained from IRC showed nonphysical imaginary frequencies after DFT optimization. Instead, a reactant monomer with the same geometry (RMSD = 0.09 Å) was used here.

[c] The imaginary frequencies were likely to be caused by numerical noise, which should not influence the tunneling coefficient and relative energy calculations significantly.

S6 Supporting data for the AceO system

S6.1 Energy barriers of post-β-scission processes

We propose that, on the triplet surface, from the β-scission reaction of ^{3}(AceO$\cdots$OAce) to the formation of acetonyl acetate ($^3CH_3C(O)OCH_2C(O)CH_3$, or $^3(C_5H_8O_3)$) include the following three steps:

1. $^3(\text{AceO}\cdots\text{OAce}) \xrightarrow{\beta-\text{scission}} {}^3(\text{CH}_3\text{C(O)}\cdots\text{CH}_2\text{O}\cdots\text{OAce})$
2. $^3(\text{CH}_3\text{C(O)}\cdots\text{CH}_2\text{O}\cdots\text{OAce}) \rightarrow {}^3(\text{CH}_3\text{C(O)}\cdots\text{OAce}) + \text{CH}_2\text{O}$
3. $^3(\text{CH}_3\text{C(O)}\cdots\text{OAce}) \xrightarrow{\text{recombination}} {}^3\text{CH}_3\text{C(O)OCH}_2\text{C(O)CH}_3$

We then constructed the key conformers in Step 3, optimized their structures at ωB97X-D3 (Grid3) level of theory. Relative energy values with respect to $^3(CH_3C(O)\cdots OAce)$ are listed in Table S11.

Table S11 Relative energies of key conformers of Step 3 in kcal mol^{-1}. Notations are defined in Table S1.

Species	ΔE_{el}(DFT)	ΔE_{elzc}(DFT)	ΔG(DFT)
$^3(CH_3C(O)\cdots OAce)$	0.00	0.00	0.00
TS of recombination	17.44	19.20	21.21
$^3(C_5H_8O_3)$	-11.55	-7.78	-5.46

S6.2 Comparison between the TS H-shift conformers reported in this and previous studies

To compare the best (G_{min}) TS conformer of the intermolecular H-shift reaction in ^{3}(AceO$\cdots$OAce), we computed the energy difference between the conformer identified by our conformer sampling workflow and the one reported by Hasan et al.[11]. The energy difference values were calculated using Equation 7:

$$\Delta E = E(\text{TS (Hasan et al.)}) - E(\text{TS } (G_{\text{min}})) \quad (7)$$

where E refers to energy terms defined in Table S1, including E_{el}, E_{elzc} and G. The initial structure of "TS (Hasan et al.)" was extracted from the publication, and then optimized at the ωB97X-D3/ma-def2-TZVP (Grid3) level.

The ΔE values are reported in kcal mol^{-1} in Table S12, as well as the imaginary frequencies in cm^{-1}.

Table S12 Relative energy values and imaginary frequencies of the best (G_{min}) TS H-shift conformers for the AceO system suggested by two studies. Notations are defined in Table S1.

Source of conformer	ΔE_{el}(DFT)	ΔE_{elzc}(DFT)	ΔG(DFT)	ω_{i}(DFT)
This study	0.00	0.00	0.00	−727.37
Study by Hasan et al.[11]	2.58	2.05	1.58	−1277.65

S7 Supporting data for the β-ISOPO system

S7.1 Energy barriers of possible reaction channels

As discussed in Section 3.2 of the main text, we computed the energy barriers of five possible pathways of the β-ISOPO system (see Figure 3) before applying the systematic conformer sampling workflow. Relevant conformers were optimized at the ωB97X-D3 (Grid3) level of theory, and the energy barriers were calculated using the following equation:

$$\Delta_b E = E_{\mathrm{TS}} - E_{\mathrm{Reactant}} \tag{8}$$

where E refers to energy terms including E_{el}, E_{elzc} and G. For the three β-scission reactions ($\mathrm{R_m}$3.2.1 – $\mathrm{R_m}$3.2.3 in Figure 3), the same reactant monomer was selected to compute the energy barriers. Similarly, the same reactant dimer was selected for the two double bond addition reactions (R3.2.2 (a) and R3.2.2 (b) in Figure 3).

Table S13 Energy barriers of possible reaction pathways for the β-ISOPO system, reported in kcal mol^{-1}. Notations are defined in Table S1.

Reaction	$\Delta_b E_{\mathrm{el}}$(DFT)	$\Delta_b E_{\mathrm{elzc}}$(DFT)	$\Delta_b G$(DFT)
$\mathrm{R_m}$3.2.1 (β-scission from the hydroxymethyl side)	5.46	3.89	3.70
$\mathrm{R_m}$3.2.2 (β-scission from the methyl side)	14.31	11.99	11.73
$\mathrm{R_m}$3.2.3 (β-scission from the ethenyl side)	25.98	22.78	21.96
R3.2.2 (a) (double bond addition at the β position)	8.35	9.10	10.79
R3.2.2 (b) (double bond addition at the γ position)	8.03	8.72	10.29

S7.2 Hydrogen bonds in isolated β−ISOPO˙ and in the complex

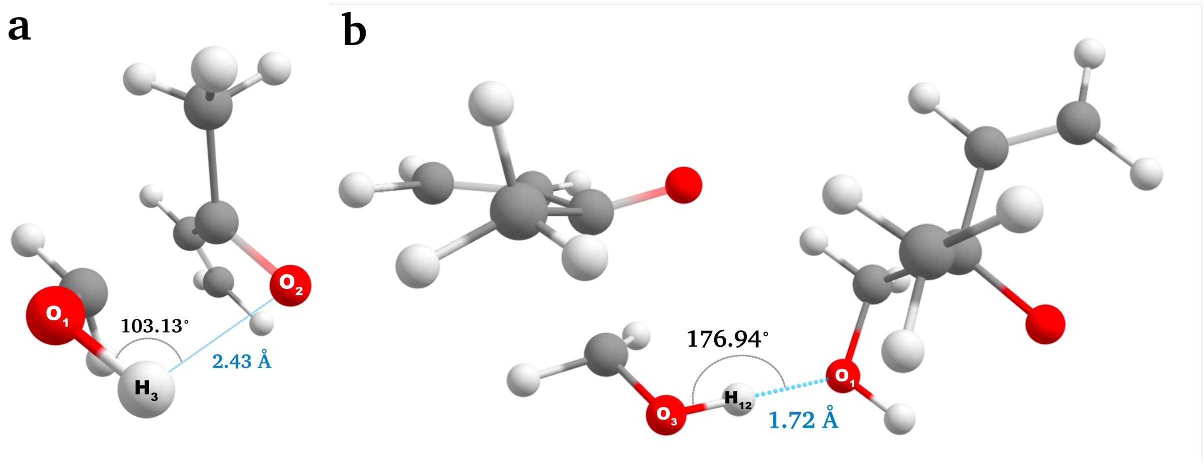

Figure S2 The TS monomer (a) and dimer (b) of the β-ISOPO system for the β-scission reaction from the hydroxymethyl side. Both 3D structures presented here are conformers with the lowest G after the ωB97X-D3 (Grid3) optimization and filtering. Color coding: gray for C, white for H, and red for O.

From Figure S2, the hydrogen bond in the TS monomer shows $d(O_2 \cdots H_3)$ = 2.426 Å, $\angle O_1H_3O_2$ = 103.13°. On the other hand, the hydrogen bond in the TS dimer shows $d(O_1 \cdots H_{12})$ = 1.716 Å, $\angle O_3H_{12}O_1$ = 176.94°. Based on their geometries, the hydrogen bond in the TS dimer is stronger than that in the TS monomer.

References

[1] T. J. Lee and P. R. Taylor, *International Journal of Quantum Chemistry*, 1989, **36**, 199–207.

[2] F. Jensen, *Introduction to computational chemistry*, John wiley & sons, 2017.

[3] R. Izsák, A. V. Ivanov, N. S. Blunt, N. Holzmann and F. Neese, *Journal of Chemical Theory and Computation*, 2023, **19**, 2703–2720.

[4] W. Jiang, N. J. DeYonker and A. K. Wilson, *Journal of chemical theory and computation*, 2012, **8**, 460–468.

[5] A. Karton, E. Rabinovich, J. M. L. Martin and B. Ruscic, *The Journal of Chemical Physics*, 2006, **125**, 144108.

[6] A. Karton, S. Daon and J. M. Martin, *Chemical Physics Letters*, 2011, **510**, 165–178.

[7] P. Cassam-Chenaï, *Theoretical Chemistry Accounts*, 2015, **134**, 125.

[8] S. Goel and A. E. Masunov, Computational Science – ICCS 2009, Berlin, Heidelberg, 2009, pp. 141–150.

[9] F. Neese *et al.*, *ORCA 6.0 Manual Section 7.6: Choice of Initial Guess and Restart of SCF Calculations*, 2024, `https://www.faccts.de/docs/orca/6.0/manual/contents/detailed/initguess.html`, Accessed March 2026.

[10] A. C. Vaucher and M. Reiher, *Journal of chemical theory and computation*, 2017, **13**, 1219–1228.

[11] G. Hasan, V.-T. Salo, R. R. Valiev, J. Kubecka and T. Kurtén, *The Journal of Physical Chemistry A*, 2020, **124**, 8305–8320.
